# Accuracy enhancement of metabolic index-based blood glucose estimation with a screening process for low-quality data

**DOI:** 10.1117/1.JBO.29.10.107001

**Published:** 2024-10-25

**Authors:** Tomoya Nakazawa, Keiji Morishita, Anna Ienaka, Takeo Fujii, Masaki Ito, Fumie Matsushita

**Affiliations:** a Hamamatsu Photonics K.K., Electron Tube Division, Shimokanzo, Iwata, Japan; b Hamamatsu Photonics K.K., Intellectual Property Headquarters, Hamamatsu, Japan

**Keywords:** non-invasive blood glucose measurement, near-infrared spectroscopy, accuracy improvement, algorithm

## Abstract

**Significance:**

Many researchers have proposed various non-invasive glucose monitoring (NIGM) approaches using wearable or portable devices. However, due to the limited capacity of detectors for such compact devices and the movement of the body during measurement, the precision of the acquired data frequently diminishes, which can cause problems during actual use in daily life. In addition, intensive smoothing is often used in post-processing to mitigate the effects of erroneous values. However, this requires a considerable amount of data and results in a delay in the response to the actual blood glucose level (BGL).

**Aim:**

Instead of just applying data smoothing in the post-process of the data acquisition, we propose an active low-quality data screening method in the pre-process. In the proposal phase of the screening process, we employ an analytical approach to examine and formulate factors that might affect the BGL estimation accuracy.

**Approach:**

A signal quality index inspired by the standard deviation concept is introduced to detect visually apparent noise on signals. Furthermore, the total estimation error in the metabolic index (MI) is calculated based on potential perturbations defined by the signal-to-noise ratio (SNR) and the uncertainty due to discrete sampling. Thereafter, the acquired data were screened by these quality indices.

**Results:**

By applying the proposed data screening process to the data obtained from a commercially available smartwatch device in the pre-process, the estimation accuracy of the MI-based BGL was improved significantly.

**Conclusions:**

Adopting the proposed screen process improves BGL estimation accuracy in the smartwatch-based prototype. Applying the proposed screen process will facilitate the integration of wearable and continuous BGL monitoring into size- and SNR-limited devices such as smartwatches and smart rings.

## 1 Introduction

To date, there is no curative treatment for type 1 and type 2 diabetes.^1^^,^^2^ Therefore, patients must monitor their blood glucose levels (BGLs) to prevent further progression of the disease. Continuous glucose monitoring (CGM) is becoming more widely used among diabetic patients,^3^ and its accuracy is steadily improving.^4^ In addition, its availability is expected to further increase since the United States Food and Drug Administration (FDA) recently granted the first over-the-counter (OTC) approval for a specific model.

Having said that, conventional self-monitoring blood glucose (SMBG) is still widely used as the first step in BGL control because of its reliability, lower cost, accuracy,^5^ and accessibility as an OTC device. However, SMBG requires a painful finger prick, which carries the risk of infection^6^^,^^7^ and sometimes results in low patient adherence.^8^

To address these problems with conventional SMBGs, the authors proposed a non-invasive glucose monitoring (NIGM) method, called the metabolic index (MI) method, which is based on the phase delay between oxy- and hemoglobin pulsation signals induced by oxygen consumption in cell respiration.^9^ Although a smartwatch-based prototype successfully demonstrated the basic idea of the proposed phase-delay-based method, which showed a proper correlation with the reference BGLs in the case of sugary and non-sugary oral challenges, the smartwatch-based prototype was not suitable enough for accurate BGL estimation at that time. Therefore, another prototype based on a smartphone camera was introduced for the repeatability test.

However, although the smartphone camera-based prototype is acceptable as a portable device, it is difficult for continuous use in daily life. Given the reported effectiveness of CGM devices for glycemic control,^10^^,^^11^ NIGM should preferably be integrated into portable devices and continuously monitor BGLs.

The main difficulties in using wearable smartwatches as NIGM devices are as follows. First, the size of the active area of the detector and sensitivity are limited,^12^ which may result in poor signal quality depending on the peripheral perfusion status.^13^ Second, smartwatches and smart rings are worn close to the extremities of the human body so that their longer moment arms can easily suffer from signal distortion and artifacts caused by body motion.^14^ These are serious obstacles not only for NIGM but also for heart rate (HR) and heart rate variability (HRV) monitoring. To address these difficulties that inevitably accompany photoplethysmography (PPG) techniques, many researchers have already proposed methods to evaluate PPG signal quality and to provide measures to mitigate low-quality data. Elgendi^15^ compared the performance of several signal quality indices (SQIs) for PPG signals previously proposed by other researchers and concluded that the skewness index showed better performance in discriminating between excellent and other lower-quality signals. Although the basic idea of the skewness index can also be applied to the NIGM, details need to be modified and optimized to fit into the MI method. A combination of inertial sensors with PPG sensors has also been proven to detect and remove motion artifacts. Wood and Asada^16^ and Lee et al.^17^ proposed motion artifact cancellation methods using accelerometers and gyroscopes, respectively. Although this type of solution would be best suited for traditional smart devices that include multiple inertial sensors for activity tracking and gesture recognition, this method can sometimes lead to high power consumption.^18^

To overcome the above concern, Tabei et al.^19^ and Afandizadeh Zargari et al.,^18^ respectively, investigated machine learning (ML)-based methods to detect and remove motion and noise artifacts (MNAs) without using accelerometers, and both achieved successful results. However, ML methods typically require a sufficient amount of data over a wide range of personal variations, including gender, age, and race. This process of data accumulation also requires a significant amount of time and money, which can sometimes be burdensome for small organizations and may prohibit the advancement of technology.

Obviously, the signal-to-noise ratio (SNR) is also crucial for the PPG signal quality. However, the effect of SNR on the accuracy of BGL estimation in the MI method has not been analyzed.

For the above reasons, a simple and specialized index to detect and reject motion artifacts for the MI method, which does not require additional inertial sensors and ML models, needs to be discussed to realize portable and continuous MI-based NIGM devices. Taking the form factor of wearable devices such as smartwatches into consideration shows that conserving battery power is essential for prolonging the lifespan of the NIGM function. Moreover, the increasing penetration rate of smartwatches across various regions and age groups, along with the ever-increasing spread of diabetes throughout the world, clearly shows that a signal quality method that is easy to implement and apply regardless of personal and individual differences is absolutely essential for meeting the increasing worldwide demand for NIGM devices. In this study, the authors first explain the SQIs for the MI method. Then, the proposed quality indexes and data rejection method are further validated through multiple oral challenge tests using a smartwatch-based prototype.

## 2 Theory and Formulation

### 2.1 Basic Formulas of the MI-Based NIGM Method

**Fig. 1:**
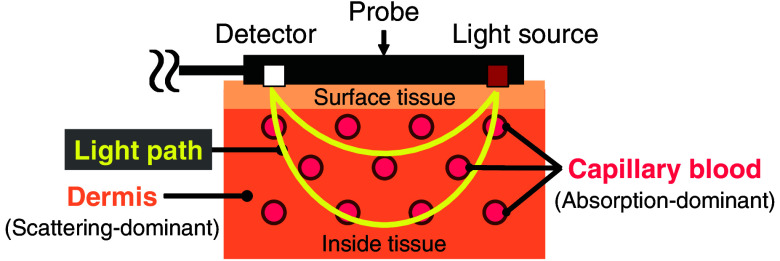
Schematic of NIRS measurement.

Figure 1 shows a schematic diagram of a near-infrared spectroscopy (NIRS) measurement on a living body. According to the modified Beer-Lambert law (MBLL), by using two different probe wavelengths and solving for the matrix calculation, changes in oxy- and deoxyhemoglobin NIRS signals $\Delta N_{{\mathrm{HbO}}_{2}}(t)$ and $\Delta N_{\mathrm{Hb}}(t)$ can be expressed as follows:^20^^–^^24^

$$\Delta N_{{\mathrm{HbO}}_{2}}(t)=\Delta (c_{{\mathrm{HbO}}_{2}}(t)\cdot L(t))=c_{{\mathrm{HbO}}_{2}}(t)\cdot L(t)-c_{{\mathrm{HbO}}_{2}}(t_{0})\cdot L(t_{0}),$$ (1)

$$\Delta N_{\mathrm{Hb}}(t)=\Delta (c_{\mathrm{Hb}}(t)\cdot L(t))=c_{\mathrm{Hb}}(t)\cdot L(t)-c_{\mathrm{Hb}}(t_{0})\cdot L(t_{0}),$$ (2)

where $c_{{\mathrm{HbO}}_{2}}(t)$ and $c_{\mathrm{Hb}}(t)$ are the molar concentrations of oxyhemoglobin and deoxyhemoglobin in the blood at time t, L(t) is the optical path length with respect to the time t, and the subscript 0 represents the initial condition, respectively.

Here, both oxy- and deoxyhemoglobin incremental NIRS signals $\Delta N_{{\mathrm{HbO}}_{2}}(t)$ and $\Delta N_{\mathrm{Hb}}(t)$ can be decomposed into quasi-DC and AC components as follows:

$$\Delta N_{{\mathrm{HbO}}_{2}}(t)=\Delta N_{{\mathrm{HbO}}_{2},\mathrm{DC}}(t)+\Delta N_{{\mathrm{HbO}}_{2},\mathrm{AC}}(t),$$ (3)

$$=[c_{{\mathrm{HbO}}_{2},\mathrm{DC}}(t)+c_{{\mathrm{HbO}}_{2},\mathrm{AC}}(t)]\cdot [L_{\mathrm{DC}}(t)+L_{\mathrm{AC}}(t)]-c_{{\mathrm{HbO}}_{2}}(t_{0})\cdot L(t_{0}),$$ (4)

$$\Delta N_{\mathrm{Hb}}(t)=\Delta N_{\mathrm{Hb},\mathrm{DC}}(t)+\Delta N_{\mathrm{Hb},\mathrm{AC}}(t),$$ (5)

$$=[c_{\mathrm{Hb},\mathrm{DC}}(t)+c_{\mathrm{Hb},\mathrm{AC}}(t)]\cdot [L_{\mathrm{DC}}(t)+L_{\mathrm{AC}}(t)]-c_{\mathrm{Hb}}(t_{0})\cdot L(t_{0}).$$ (6)

**Fig. 2:**
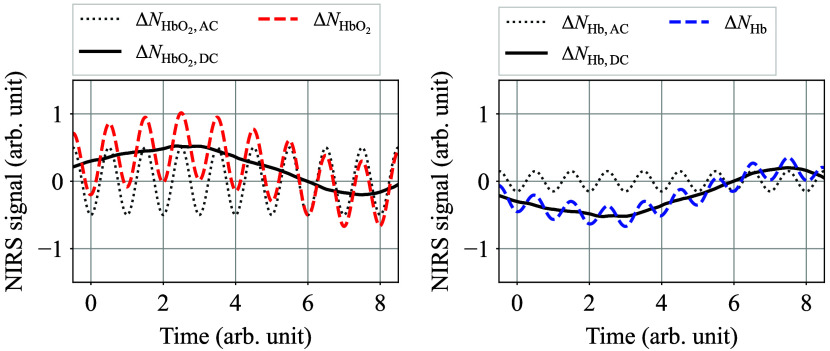
Conceptual diagram of NIRS signal decomposition into quasi-DC and AC components.

Here, the subscripts AC and DC indicate the AC and quasi-DC components of the corresponding physical quantity, respectively. For reference, a visual explanation of this decomposition operation is shown in Fig. 2.

**Fig. 3:**
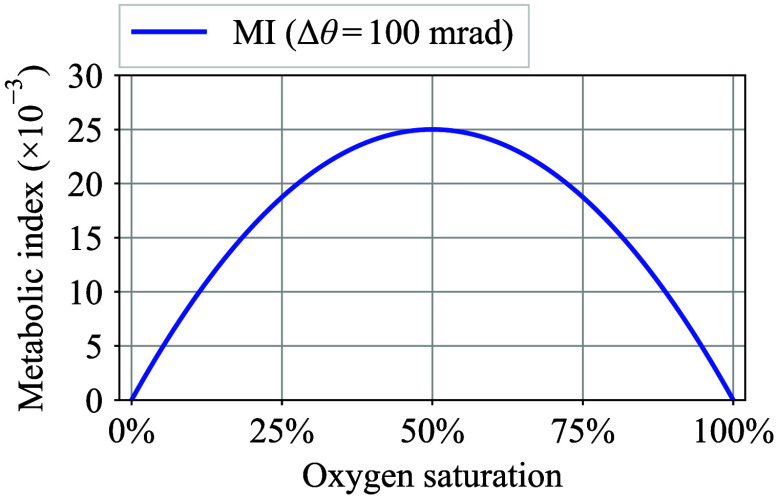
Metabolic index MI behavior versus oxygen saturation.

Here, by applying several assumptions and approximations, the oscillation amplitude of the AC component of deoxyhemoglobin concentration $C_{\mathrm{Hb},\mathrm{AC}}(t)$ is obtained as follows:^9^

$$C_{\mathrm{Hb},\mathrm{AC}}(t)\approx C_{0}\cdot \frac{L_{\mathrm{AC},\text{amplitude}}(t)}{L(t)}\cdot \mathrm{MI}(t),$$ (7)

where $C_{0}$ is the total hemoglobin concentration, $L_{\mathrm{AC},\text{amplitude}}(t)$ is the oscillation amplitude of the AC component of the optical path length $L_{\mathrm{AC}}(t)$, and MI(t) is a dimensionless metabolic index defined as follows:

$$\mathrm{MI}(t)={\mathrm{SaO}}_{2}(t)\cdot [1-{\mathrm{SaO}}_{2}(t)]\cdot |\Delta \theta (t)|,$$ (8)

where Δθ is the tiny phase delay between oxy- and deoxyhemoglobin signals $\Delta N_{{\mathrm{HbO}}_{2},\mathrm{AC}}(t)$ and $\Delta N_{\mathrm{Hb},\mathrm{AC}}(t)$, and ${\mathrm{SaO}}_{2}(t)$ is the arterial oxygen saturation, which can be approximated as follows:

$${\mathrm{SaO}}_{2}(t)\approx \frac{\Delta N_{{\mathrm{HbO}}_{2},\mathrm{AC},\text{amplitude}}(t)}{\Delta N_{{\mathrm{HbO}}_{2},\mathrm{AC},\text{amplitude}}(t)+\Delta N_{\mathrm{Hb},\mathrm{AC},\text{amplitude}}(t)},$$ (9)

where $\Delta N_{{\mathrm{HbO}}_{2},\mathrm{AC},\text{amplitude}}(t)$ and $\Delta N_{\mathrm{Hb},\mathrm{AC},\text{amplitude}}(t)$ are the amplitudes of the AC components of the oxy- and deoxyhemoglobin signals, respectively. To help visually comprehend Eq. (8), Fig. 3 illustrates the behavior of the MI versus oxygen saturation when Δθ=100  mrad. As can be seen in Fig. 3, MI increased along with a decrease in the oxygen saturation from 100% and reached a maximum value at 50% saturation. Conversely, MI becomes zero at 100% oxygen saturation. This behavior suggests that the metabolic activity resulting from local cell respiration occurs concurrently with a lower peripheral oxygen saturation that is attributable to oxygen transactions in capillaries.

Here, by applying the assumption about the relationship between L(t) and $L_{\mathrm{AC},\text{amplitude}}(t)$, Eq. (7) can be rewritten as follows:^9^

$$C_{\mathrm{Hb},\mathrm{AC}}(t)\approx C_{0}\cdot \frac{L_{\mathrm{AC},\text{amplitude},0}}{L_{\mathrm{DC},0}}\cdot \alpha (t)\cdot \mathrm{MI}(t),$$ (10)

$$=C_{0}\cdot \frac{L_{\mathrm{AC},\text{amplitude},0}}{L_{\mathrm{DC},0}}\cdot MI^{\prime }(t),$$ (11)

where

$$MI^{\prime }(t)=\alpha (t)\cdot \mathrm{MI}(t).$$ (12)

Here, $L_{\mathrm{AC},\text{amplitude},0}$ and $L_{\mathrm{DC},0}$ are AC and quasi-DC components of the optical path length in the initial condition, respectively. The term $MI^{\prime }(t)$ denotes an amplitude-corrected metabolic index, and α(t) denotes a dimensionless optical path length correction factor, expressed as follows:^9^

$$\alpha (t)={\left[\frac{L_{\mathrm{AC},\text{amplitude}}(t)}{L_{\mathrm{AC},\text{amplitude},0}}\right]}^{1-\frac{1}{n}}\;(0<n\leq 1).$$ (13)

Here, $L_{\mathrm{AC}}(t)$ can be obtained from $\Delta N_{{\mathrm{HbO}}_{2},\mathrm{AC},\text{amplitude}}(t)$ and $\Delta N_{\mathrm{Hb},\mathrm{AC},\text{amplitude}}(t)$ by employing the following relationship:

$$L_{\mathrm{AC},\text{amplitude}}(t)=\frac{1}{C_{0}}[\Delta N_{{\mathrm{HbO}}_{2},\mathrm{AC},\text{amplitude}}(t)+\Delta N_{\mathrm{Hb},\mathrm{AC},\text{amplitude}}(t)].$$ (14)

As $C_{\mathrm{Hb},\mathrm{AC}}(t)$ corresponds to the oxygen consumption in each cardiac cycle of the probed region and BGL affects the living body metabolism, $C_{\mathrm{Hb},\mathrm{AC}}(t)$ can be assumed to exhibit a strong correlation with BGL. Furthermore, given $C_{0}$, $L_{\mathrm{AC},\text{amplitude},0}$, and $L_{\mathrm{DC},0}$ are all constant, BGL can be estimated by monitoring $MI^{\prime }(t)$.

### 2.2 Derivation of the Signal Quality Index for the MI Method

**Fig. 4:**
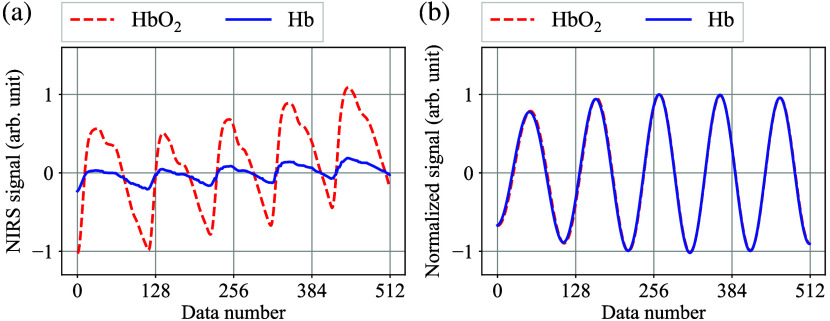
(a) Typical example of the sampled oxy- and deoxyhemoglobin waveforms. (b) Normalized BPF-applied waveforms of panel (a).

To evaluate the signal quality for the AC oxy- and deoxy hemoglobin NIRS signals $\Delta N_{{\mathrm{HbO}}_{2},\mathrm{AC}}(t)$ and $\Delta N_{\mathrm{Hb},\mathrm{AC}}(t)$, the standard deviation concept is introduced. Figure 4(a) shows a typical example of the discretely-sampled oxy- and deoxyhemoglobin NIRS signals, and Fig. 4(b) illustrates the band-pass-filter (BPF)-applied and normalized waveforms of Fig. 4(a), where components around the HR frequency are extracted.

As can be seen in Fig. 4(b), the two waveforms exhibit a high degree of similarity with minimal discernible differences in ideal conditions. In this study, the authors sought to quantify the dissimilarity between the two normalized BPF-applied waveforms by employing the standard deviation s(t), which is defined as

$$s(t)=\sqrt{\frac{1}{l}\sum_{k=1}^{l}(p_{k}-q_{k}{)}^{2}},$$ (15)

where l is the discretely sampled data length and $p_{k}$ and $q_{k}$ are the elements of normalized BPF-applied oxy- and deoxyhemoglobin data, respectively. Given that $p_{k}$ and $q_{k}$ are BPF-applied values, the standard deviation s is nearly zero under regular conditions.

**Fig. 5:**
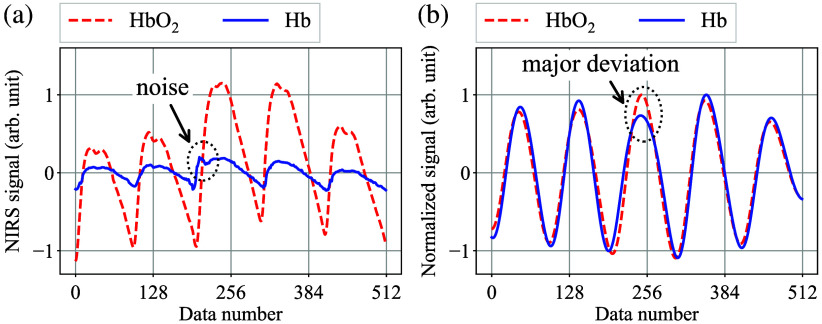
(a) Example of the sampled oxy- and deoxyhemoglobin waveforms with noise. (b) Normalized BPF-applied waveforms of (a).

Here, consider the scenario where noise is added to the NIRS waveforms. Figure 5(a) shows an example of the discretely-sampled oxy- and deoxyhemoglobin signals with noise, and Fig. 5(b) illustrates the normalized BPF-applied waveforms of Fig. 5(a), where components around the HR frequency are extracted. In this example, a small spike is superimposed on the deoxyhemoglobin waveform around data number 200. Figure 5(b) illustrates that a minor fluctuation in the NIRS waveform can have a significant impact on the BPF-applied waveform, resulting in an increase in the standard deviation s(t).

Nevertheless, even under optimal conditions, the standard deviation s(t) is constrained by the inherent delay between the oxy- and deoxyhemoglobin NIRS waveforms, which is on the order of several tens of milliradians. Given a specific value for the phase delay Δθ(t), the reachable standard deviation limit σ(t) can be expressed as follows:

$$\sigma (t)=\sqrt{1-\cos\;\Delta \theta (t)}\leq s(t).$$ (16)

Here, the derivation process of Eq. (16) is described in detail in Sec. S1 of the Supplementary Material. Subsequently, by applying a deformation to Eq. (16), the following is derived:

$$\Delta \theta_{\sigma }(t)=\cos^{-1}[1-s^{2}(t)]\geq |\Delta \theta (t)|,\;[\Delta \theta_{\sigma }(t)\geq 0],$$ (17)

where $\Delta \theta_{\sigma }(t)$ is the estimated phase delay based on the standard deviation s(t). Equation (17) indicates that the phase delay Δθ(t) can be calculated from the standard deviation in addition to the method of comparing the fast Fourier transform (FFT) phase at the main peak of each spectrum of $\Delta N_{{\mathrm{HbO}}_{2},\mathrm{AC}}(t)$ and $\Delta N_{\mathrm{Hb},\mathrm{AC}}(t)$, which was employed in the previous research.^9^ Hereafter, for the sake of convenience, the FFT-phase-based Δθ(t) is referred to as $\Delta \theta_{\mathrm{FFT}}(t)$. Finally, the signal quality of the oxy- and deoxyhemoglobin NIRS signals can be evaluated by calculating the phase error $\varepsilon_{\sigma }(t)$, which is expressed as follows:

$$\varepsilon_{\sigma }(t)=||\Delta \theta_{\mathrm{FFT}}(t)|-\Delta \theta_{\sigma }(t)|.$$ (18)

In this study, each calculated $\Delta \theta_{\mathrm{FFT}}(t)$ is initially screened by $\varepsilon_{\sigma }(t)$ to be less than a predetermined value.

### 2.3 Derivation of Theoretical Phase Estimation Errors Defined by the Background Noise Level

Although $\varepsilon_{\sigma }(t)$ in Eq. (18) is effective in identifying and rejecting visually recognizable errors in NIRS signals, it lacks the capacity to assess potential errors that are not visually apparent. In a strict sense, the FFT-based phase delay, $\Delta \theta_{\mathrm{FFT}}$, is inherently affected by perturbation from the background noise, which is typically defined according to the noise floor level. This results in an error in phase detection. In this subsection, the authors attempt to formulate the phase estimation error defined by SNR.

**Fig. 6:**
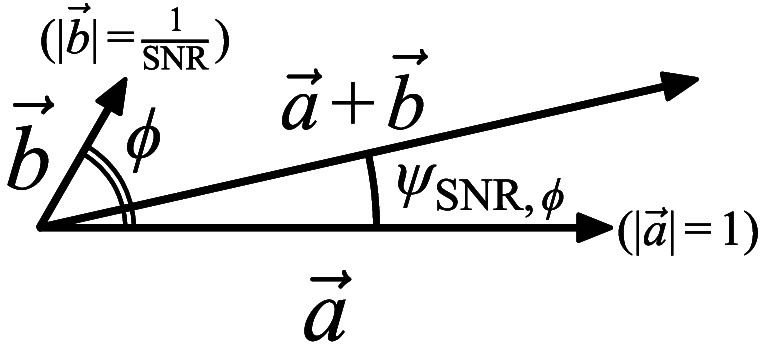
Basic concept of the phase estimation error caused by the background noise.

Figure 6 illustrates the basic idea of the phase estimation error caused by the random background noise. Here, $\vec{a}$ represents the normalized main peak vector of the FFT-applied NIRS signal, and $\vec{b}$ represents the background noise vector with amplitude and argument of 1/SNR(t) and ϕ, respectively. In this representation, the phase estimation error, which is denoted by $\psi_{\mathrm{SNR},\phi }$, can be replaced by the argument of the combined vector $\vec{a}+\vec{b}$ and can be expressed as follows:

$$\psi_{\mathrm{SNR},\phi }=\tan^{-1}(\frac{\nu \;\sin\;\phi }{1+\nu \;\cos\;\phi }),$$ (19)

where ν is the inverse value of the SNR(t), which can also be called the noise-to-signal ratio, and it is assumed that SNR(t) can be treated as a constant within a limited measurement period.

Then, the typical phase estimation error due to the background noise, denoted by $\varepsilon_{\mathrm{SNR}}(t)$, can be calculated as the square root of the mean value of $\psi_{\mathrm{SNR},\phi }^{2}$ over −π≤ϕ≤π, which is expressed as follows:

$$\varepsilon_{\mathrm{SNR}}(t)=\sqrt{\frac{1}{2\pi }\int_{-\pi }^{\pi }\psi_{\mathrm{SNR},\phi }^{2}d\phi }=\sqrt{\frac{1}{2\pi }\int_{-\pi }^{\pi }{\left[\tan^{-1}(\frac{\nu \;\sin\;\phi }{1+\nu \;\cos\;\phi })\right]}^{2}d\phi }.$$ (20)

Here, Eq. (20) cannot be solved analytically. However, by assuming that ν, the inverse value of the SNR, is sufficiently less than 1, Eq. (20) can be approximated as

$$\varepsilon_{\mathrm{SNR}}(t)\approx \frac{1}{\sqrt{2}}\cdot \frac{1}{\mathrm{SNR}(t)}.$$ (21)

The derivation process of Eq. (21) is described in detail in Sec. S2 of the Supplementary Material. Here, $\Delta \theta_{\mathrm{FFT}}(t)$ is practically calculated by comparing the FFT phase of the oxy- and deoxyhemoglobin signals at the HR frequency. Therefore, the total phase estimation error, denoted by $\varepsilon_{\mathrm{SNR}(t),\text{total}}$, should be expressed as the resultant error as follows:

$$\varepsilon_{\mathrm{SNR},\text{total}}(t)=\sqrt{\varepsilon_{\mathrm{SNR},{\mathrm{HbO}}_{2}}^{2}(t)+\varepsilon_{\mathrm{SNR},\mathrm{Hb}}^{2}(t)},$$ (22)

$$\approx \frac{1}{\sqrt{2}}\cdot \sqrt{\frac{1}{{\mathrm{SNR}}_{{\mathrm{HbO}}_{2}}^{2}(t)}+\frac{1}{{\mathrm{SNR}}_{\mathrm{Hb}}^{2}(t)}},$$ (23)

where $\varepsilon_{\mathrm{SNR},{\mathrm{HbO}}_{2}}(t)$ and $\varepsilon_{\mathrm{SNR},\mathrm{Hb}}(t)$ are FFT-phase estimation errors of the oxy- and deoxyhemoglobin NIRS signals at the HR frequency, and ${\mathrm{SNR}}_{{\mathrm{HbO}}_{2}}(t)$ and ${\mathrm{SNR}}_{\mathrm{Hb}}(t)$ are SNR of oxy- and deoxyhemoglobin NIRS signals, respectively.

Typically, the signal amplitude of the deoxyhemoglobin NIRS signal is approximately one-tenth of that of the oxyhemoglobin NIRS signal. Assuming that the background noise amplitude is almost identical between oxy- and deoxyhemoglobin NIRS signals, ${\mathrm{SNR}}_{\mathrm{Hb}}(t)$ is ∼10 times worse than ${\mathrm{SNR}}_{{\mathrm{HbO}}_{2}}(t)$. Therefore, $\varepsilon_{\mathrm{SNR},\mathrm{Hb}}(t)$ is dominant in Eq. (22), and Eq. (23) can be approximated as follows:

$$\varepsilon_{\mathrm{SNR},\text{total}}(t)\approx \frac{1}{\sqrt{2}}\cdot \frac{1}{{\mathrm{SNR}}_{\mathrm{Hb}}(t)}.$$ (24)

As indicated by Eq. (24), $\varepsilon_{\mathrm{SNR},\text{total}}(t)$ becomes as high as 70 mrad when ${\mathrm{SNR}}_{\mathrm{Hb}}(t)$ is 10, or 20 dB in decibels. In the previous research, 40  mg/dL of BGL change induced ∼50  mrad of phase delay in $\Delta \theta_{\mathrm{FFT}}(t)$.^9^ Consequently, the suboptimal quality of ${\mathrm{SNR}}_{\mathrm{Hb}}(t)$ may readily compromise the precision of the BGL estimation.

### 2.4 Derivation of Theoretical Phase Estimation Errors Defined by the Data Sampling Frequency

It is also crucial to consider the sampling frequency of the NIRS signals to estimate the phase estimation error. When the sampling frequency and the HR frequency are defined as $f_{s}$ and $f_{\mathrm{HR}}(t)$, respectively, the mathematical phase division step size of the discretely sampled NIRS signals, denoted by $\theta_{\mathrm{div}}(t)$, can be expressed as follows:

$$\theta_{\mathrm{div}}(t)=2\pi \frac{f_{\mathrm{HR}}(t)}{f_{s}}.$$ (25)

**Fig. 7:**
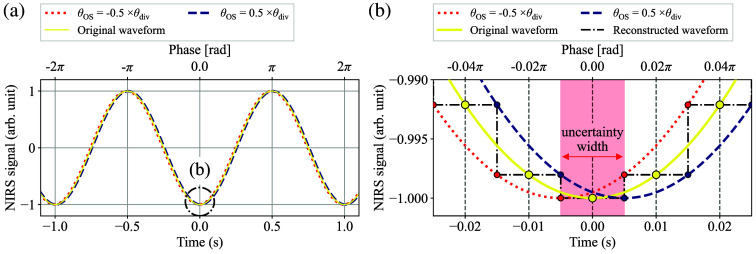
(a) Basic concept of the phase estimation error resulting from discrete sampling. (b) An enlarged view of panel (a) at t=0.

Here, Figs. 7(a) and 7(b) illustrate examples of the potential range of temporal uncertainty resulting from discrete sampling, where Fig. 7(b) is an enlarged view of Fig. 7(a) at t=0. For the sake of simplicity, the values of $f_{s}$ and $f_{\mathrm{HR}}(t)$ are set to 100 and 1.0 Hz in Figs. 7(a) and 7(b). In addition, the term $\theta_{\mathrm{OS}}$ represents the phase offset from the original waveform, the dashed vertical lines in Fig. 7(b) represent the horizontal sampling step size determined by $f_{s}$ and $f_{\mathrm{HR}}(t)$, and the dash-dot lines in Fig. 7(b) represent a waveform reconstructed from the discretely sampled data.

Given that the temporal phase uncertainty resulting from discrete sampling is constrained by the boundaries of $\pm \theta_{\mathrm{div}}(t)/2$, its probability can be approximated by the continuous uniform distribution. Subsequently, by applying the formula for the standard deviation of the continuous uniform distribution, the typical phase uncertainty due to discrete sampling $\varepsilon_{s}(t)$ can be expressed as follows:

$$\varepsilon_{s}(t)=\sqrt{\frac{1}{12}\theta_{\mathrm{div}}^{2}(t)}=\frac{\theta_{\mathrm{div}}(t)}{\sqrt{12}}.$$ (26)

As with the case of $\varepsilon_{\mathrm{SNR}}$, both the oxy- and deoxyhemoglobin NIRS signals have their own sampling uncertainty. Consequently, the total sampling uncertainty, denoted by $\varepsilon_{s,\text{total}}(t)$, can be given as follows:

$$\varepsilon_{s,\text{total}}(t)=\sqrt{\varepsilon_{{s,\mathrm{HbO}}_{2}}^{2}(t)+\varepsilon_{s,\mathrm{Hb}}^{2}(t)}=\sqrt{2}\cdot \varepsilon_{s}(t)=\frac{\theta_{\mathrm{div}}(t)}{\sqrt{6}}.$$ (27)

### 2.5 Total Estimation Error of the Metabolic Index Resulting from Phase Estimation Errors

From Eqs. (8), (10), (11), (24), and (27), the combined value of the latent estimation errors of the metabolic index $MI^{\prime }(t)$, denoted by $\delta MI^{\prime }(t)$, can be approximated as follows:

$$\delta MI^{\prime }(t)\approx \alpha (t)\cdot {\mathrm{SaO}}_{2}(t)\cdot [1-{\mathrm{SaO}}_{2}(t)]\cdot \varepsilon_{\text{total}}(t),$$ (28)

$$\varepsilon_{\text{total}}(t)=\sqrt{\varepsilon_{\mathrm{SNR},\text{total}}^{2}(t)+\varepsilon_{s,\text{total}}^{2}(t)}\approx \sqrt{\frac{1}{2}\cdot \frac{1}{{\mathrm{SNR}}_{\mathrm{Hb}}^{2}(t)}+\frac{\theta_{\mathrm{div}}^{2}(t)}{6}},$$ (29)

where $\varepsilon_{\text{total}}(t)$ is the resultant error of $\varepsilon_{\mathrm{SNR},\text{total}}(t)$ and $\varepsilon_{s,\text{total}}(t)$. Here, the estimation errors of α(t) and ${\mathrm{SaO}}_{2}(t)$ resulting from the background noise are ignored for having limited impacts on $MI^{\prime }(t)$ compared with the phase estimation errors introduced in this paper.

In this study, the value of $\Delta \theta_{\mathrm{FFT}}(t)$ screened by $\varepsilon_{\sigma }(t)$ in Eq. (18) is subjected to further examination based on $\delta MI^{\prime }(t)$, to ensure the estimation quality.

## 3 Materials and Methods

This section presents an examination of a proposed low-quality data rejection method utilizing a two-stage screening process. The examination is conducted through a series of oral challenge tests using a smartwatch-based prototype device.

### 3.1 Smartwatch-Based Prototype

**Fig. 8:**
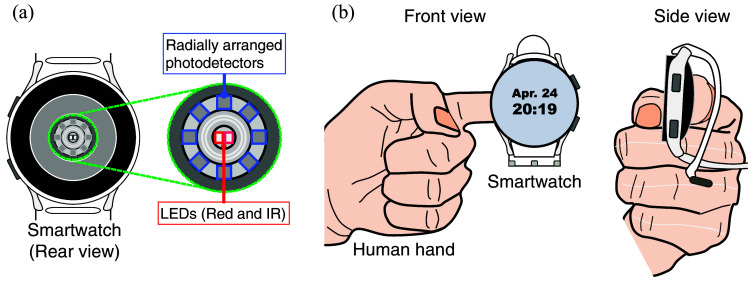
(a) Schematic rear view of the smartwatch experimental unit. (b) A schematic diagram of the application style of the smartwatch prototype on a fingertip.

In this study, the Samsung Galaxy Watch 4 44 mm (SM-R870) was utilized as the experimental unit in a manner consistent with that employed in the previous research.^9^ Figure 8(a) shows the schematic rear view of the SM-R870. Red and infrared (IR) LEDs are located in the center, and their center wavelengths are ∼650 and 930 nm, respectively. Eight photodetectors are arranged radially around the LEDs. The sampling frequency of the red and IR LED signals is 100 Hz. Here, no hardware modifications were made to the prototype, and the BGL estimation function was implemented in software. Figure 8(b) shows a schematic of the application style of the prototype device. Here, the device was wrapped around a finger pad by taking account of the capillary density of the measurement portion, which correlates strongly with signal quality.

#### 3.1.1 Experimental protocol

**Fig. 9:**
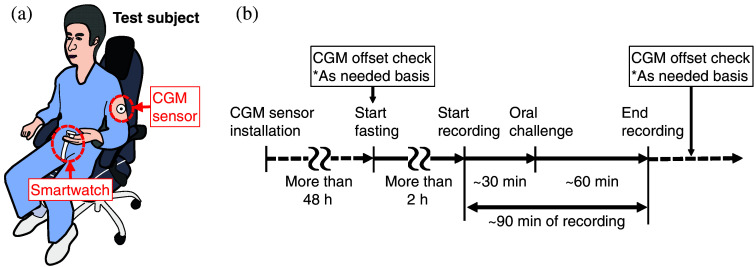
(a) Schematic diagram of the clinical trial setup. (b) Clinical trial procedure.

Figure 9(a) shows the schematic of the clinical test setup. A protocol was established while referencing factors that can affect reading values in the case of a pulse oximeter.^25^ To ensure consistency in the acquired data, a single, healthy, and non-diabetic male was selected as the sole test subject. The test subject was asked to sit still during the experiment with the smartwatch resting on their finger pad to reduce fluctuations in peripheral blood flow caused by minor changes in posture and local blood pressure. The subject was also asked to rest his elbows on armrests. In addition, before starting the measurement, the subject held a hand warmer for a few minutes to ensure adequate peripheral blood flow.

Figure 9(b) shows the typical course of a clinical test. A CGM device (Abbot, FreeStyle Libre^®^), which records BGL values at 15-min intervals, was used as a reference for the BGLs. To use CGM sensors at their best performance, CGM sensors were installed at least two days before the experiments for aging.^26^ Furthermore, the sensors were not utilized for clinical testing during the final two days of their specified expiration period. In addition, to correct for the systematic offset and delay specific to each CGM sensor, SMBG strips (Abbot, FreeStyle Precision^®^, Chicago, Illinois, United States) were used as needed before and after data recording by the smartwatch. To prevent possible distortion of the PPG signal due to body movements resulting from SMBG usage, no SMBG strips were used during the recording period. Data recording then started after at least 2 h of fasting, oral challenges were given ∼20 to 30 min after the start of recording, and data recording continued ∼60  min after the oral challenges until the subject’s BGL had mostly returned to the initial level. As a result, ∼90  min of PPG data were collected in each data recording. The clinical tests were conducted over a period of four months, with a total of 30 repeatability tests performed.

In this research, sugar-containing carbonated beverages and glucose-containing jelly beverages were used for oral challenges. For reference, Table S1 in Sec. S3 of the Supplementary Material shows the main nutritional values of the oral challenges. When administering oral challenges, care was taken to ensure that all oral challenges were not excessively cold, thus avoiding peripheral blood flow reduction due to the lowering of the body temperature.

All clinical trials described in this paper were conducted in accordance with the Clinical Trials Act of the Ministry of Health, Labor and Welfare of Japan, published on the basis of the Declaration of Helsinki, and were approved by the Ethical Committee of Hamamatsu Photonics K. K. Informed consent was obtained from the subject before measurements were performed. All clinical tests were performed under the supervision of the co-author having a medical license.

#### 3.1.2 Data processing

**Fig. 10:**
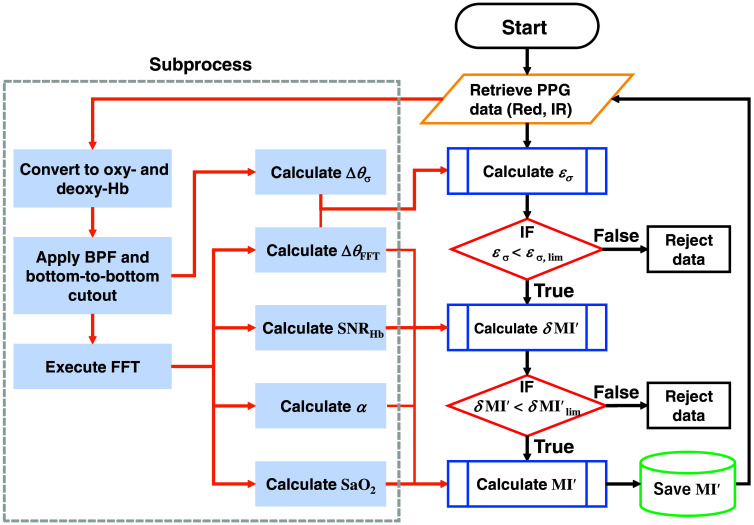
Process flowchart of the modified metabolic index $MI^{\prime }(t)$ calculation and screening.

Figure 10 depicts the flowchart for the calculation and data screening process of the modified metabolic index $MI^{\prime }(t)$ based on the data quality metrics mentioned in Eqs. (18) and (28). Here, $\varepsilon_{\sigma ,\lim}$ and $\delta {MI^{\prime }}_{\lim}$ represent arbitrary-defined thresholds for $\varepsilon_{\sigma }(t)$ and $\delta MI^{\prime }(t)$, respectively.

Initially, the retrieved data underwent preprocessing. Raw PPG data from red and IR LEDs were retrieved and accumulated to a certain data length. Then, the raw PPG data were converted to the incremental NIRS signals $\Delta N_{{\mathrm{HbO}}_{2}}(t)$ and $\Delta N_{\mathrm{Hb}}(t)$. Furthermore, BPF was then applied to each NIRS signal to remove unnecessary high-frequency components and low-frequency components due to respiratory cycles. Here, two different BPF parameter sets were used for different purposes. One parameter set was for $\Delta \theta_{\sigma }(t)$ calculation, which extracts around the HR components of NIRS signals. The other parameter set was for FFT calculation, which extracts 0.8 to 10 Hz components. After applying the BPFs, the excess end portions in each waveform were trimmed so that the first and last points of the data corresponded to the beginning and end of the pulse wave. Furthermore, FFT was applied to the filtered-and-trimmed NIRS signals. Here, each signal for FFT calculation was resampled to make the data length the smallest power of two greater than or equal to the original length. Subsequently, the BPF-applied signals and FFT spectra were employed to calculate $\Delta \theta_{\sigma }(t)$, $\Delta \theta_{\mathrm{FFT}}(t)$, ${\mathrm{SNR}}_{\mathrm{Hb}}(t)$, α(t), and ${\mathrm{SaO}}_{2}(t)$, which comprise $\varepsilon_{\sigma }(t)$, $\delta MI^{\prime }(t)$, and $MI^{\prime }(t)$. Finally, only those $MI^{\prime }(t)$ elements that satisfy the specified screening conditions were retained as valid data.

**Fig. 11:**
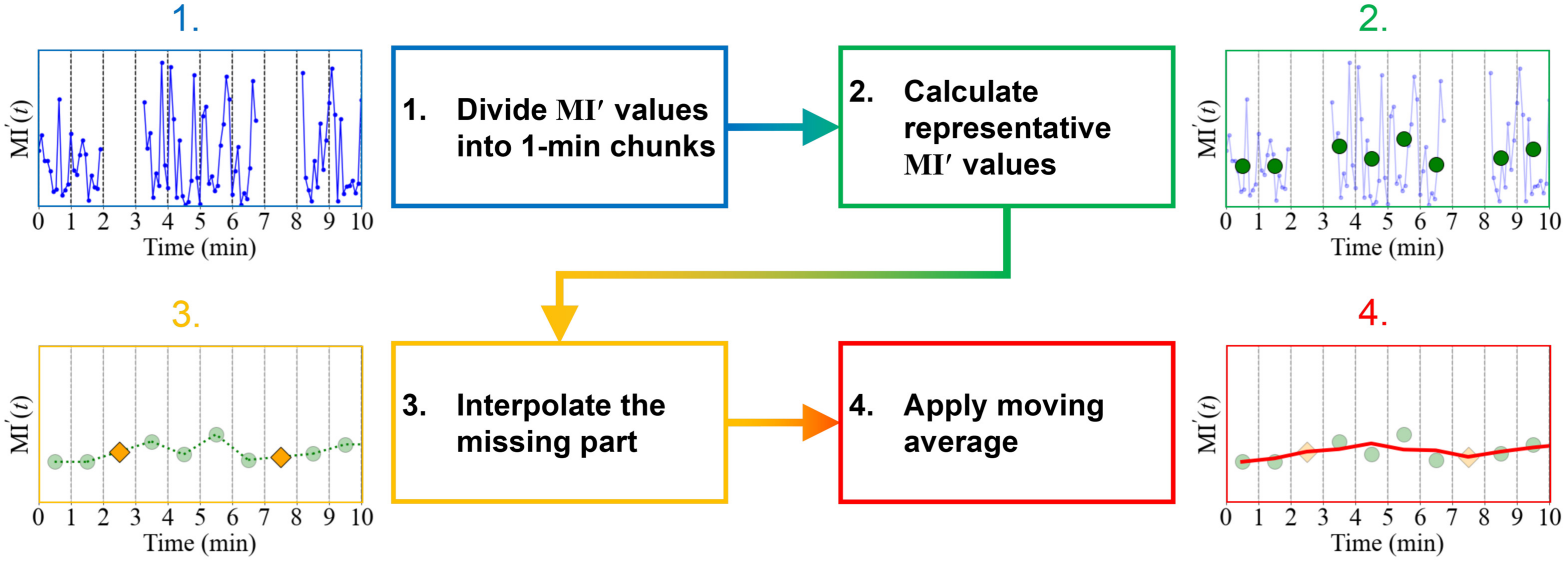
Flowchart of the postprocessing for the stored $MI^{\prime }(t)$ data.

Once the preprocessing was complete, the recorded dataset of $MI^{\prime }(t)$ was subjected to further postprocessing. Figure 11 shows a flowchart and its visual explanation of the postprocessing.

First, the $MI^{\prime }(t)$ values were divided into 1-min chunks. Second, the representative value within each chunk was calculated. Third, linear interpolation was employed to address the absence of representative values resulting from the low-quality data rejection in the preprocessing stage. Finally, the moving average was applied to the representative values. In this study, the median value was employed as the representative value for each 1-min interval, and the window length for the moving average was set at 30 points, which is equivalent to 30 min for a 1-min interval dataset. This two-stage smoothing approach is beneficial for identifying and eliminating residual outlier values, which have been shown to have a favorable correlation with the reference CGM values from previous research.^9^

### 3.2 First-Stage Screening Results for the Obtained Data

**Fig. 12:**
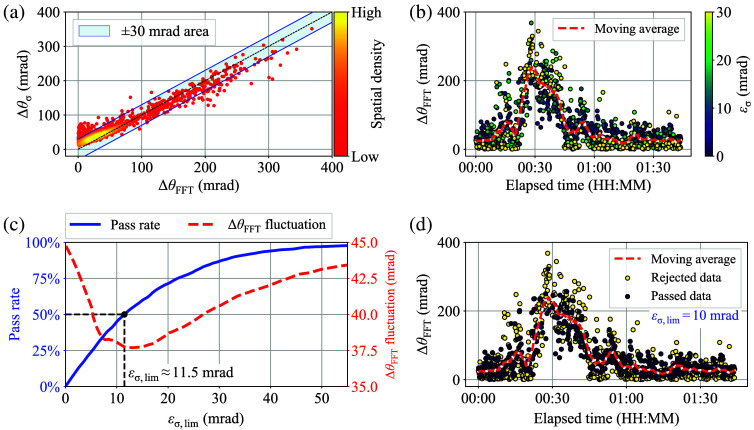
(a) Example scatter plot of $\Delta \theta_{\sigma }(t)$ versus $\Delta \theta_{\mathrm{FFT}}(t)$. (b) Time-series plot of $\Delta \theta_{\mathrm{FFT}}(t)$ generated from the data of panel (a). (c) Typical transition of the pass rate and the $\Delta \theta_{\mathrm{FFT}}$ fluctuation level according to $\varepsilon_{\sigma ,\lim}$. (d) Binarized result of panel (b) for the passed and rejected data.

Figure 12(a) depicts an example scatter plot of $\Delta \theta_{\sigma }(t)$ versus $\Delta \theta_{\mathrm{FFT}}(t)$ generated from a typical oral challenge test. Here, for illustrative purposes, the area of the ±30  mrad error region is depicted in Fig. 12(a), and the color scale in the plot indicates the spatial density of nearby points. Although the majority of data points were distributed around the diagonal line, notable amounts of outliers were confirmed. Figure 12(b) shows a time-series plot of $\Delta \theta_{\mathrm{FFT}}(t)$ generated from the same data as Fig. 12(a). Here, the color scale in each data point indicates $\varepsilon_{\sigma }(t)$, and the dashed red curve shows the smoothed values. Figure 12(b) shows that data points close to the smoothed curve have lower $\varepsilon_{\sigma }(t)$ and vice versa. This result suggests that outlier data can be excluded by examining $\varepsilon_{\sigma }(t)$. Then, an appropriate value for the acceptable $\varepsilon_{\sigma }(t)$ limit $\varepsilon_{\sigma ,\lim}$ needs to be determined. Figure 12(c) shows the typical transition of the pass rate and the $\Delta \theta_{\mathrm{FFT}}$ fluctuation level according to different $\varepsilon_{\sigma ,\lim}$ values. Here, the typical $\Delta \theta_{\mathrm{FFT}}$ fluctuation level was derived by calculating the standard deviations of the difference between adjacent time-domain $\Delta \theta_{\mathrm{FFT}}$ data after applying the $\varepsilon_{\sigma }$-screening. Although it is possible to establish strict data screening by applying a tighter $\varepsilon_{\sigma ,\lim}$, eliminating the excessive amount of data may result in the generation of inaccurate representative values in the post-processing. To achieve a balance between the two opposing factors of stricter screening and higher pass rate, 10 mrad of $\varepsilon_{\sigma ,\lim}$, which yields ∼50% of the pass rate and a quasi-minimum value in the $\Delta \theta_{\mathrm{FFT}}$ fluctuation level, has been adopted in this study. Finally, Fig. 12(d) shows the binarized result of Fig. 12(b) for the passed and rejected data when $\varepsilon_{\sigma ,\lim}=10\;\mathrm{mrad}$. With reference to Fig. 12(d), it was revealed that a sufficient amount of outlier values could be eliminated by $\varepsilon_{\sigma }$-screening while maintaining an adequate quantity of data for the post-processing. Here, it is more probable that the remaining outliers in Fig. 12(d) are the result of a random correlation caused by fluctuations in both $\Delta \theta_{\sigma }(t)$ and $\Delta \theta_{\mathrm{FFT}}(t)$. Therefore, it was not possible to eliminate them by applying a stricter threshold. For reference, Figs. S2(a)–S2(d) in the Supplementary Material show binarized results of Fig. 12(b) for various $\varepsilon_{\sigma ,\lim}$ values.

### 3.3 Post-Processed Results for the Obtained Data

**Fig. 13:**
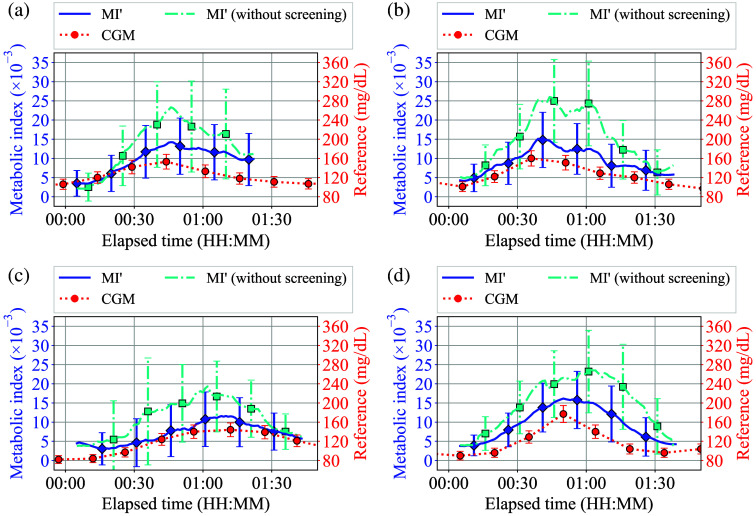
(a)–(d) Typical post-processed results of oral challenge tests.

Figures 13(a)–13(d) show typical post-processed results of oral challenge tests. For comparison, the $MI^{\prime }(t)$ evaluation result without $\delta MI^{\prime }$-screening was indicated in each plot. The CGM delays were adjusted by using the sensor-specific constant value of each sensor, which was determined through comparison with SMBG readings during the pre- or post-experimental period, and the error bars calculated using the typical values of $\delta MI^{\prime }(t)$ are indicated in each $MI^{\prime }(t)$ curve. For the reference CGM values, the 10% vertical error bar range was applied, taking into account the typical accuracy of the sensor.^4^ In addition, the ranges of the left and right vertical axes are adjusted based on the relationship between $MI^{\prime }(t)$ and BGL as established in the previous research.^9^ In this study, $\delta {MI^{\prime }}_{\lim}=10\times 10^{-3}$ of threshold and n=0.5 of power exponent in α-correction have been applied, and the optimization process will be explained later on. Looking at each of Figs. 13(a)–13(d), it can be seen that the error bar width and the overestimation of $MI^{\prime }(t)$ have been effectively mitigated through the implementation of $\delta MI^{\prime }$-screening.

**Fig. 14:**
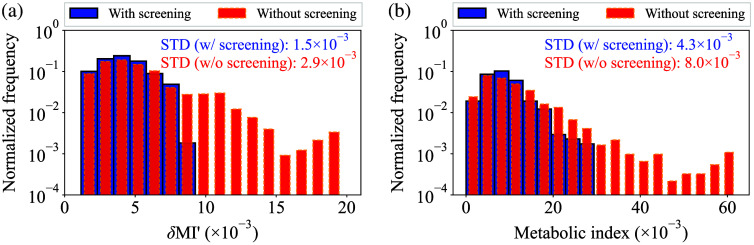
(a) Comparison of the $\delta MI^{\prime }$ histograms between the case with and without $\delta MI^{\prime }$-screening. (b) Comparison of the $MI^{\prime }$ histograms between the case with and without $\delta MI^{\prime }$-screening.

To illustrate the impact of the $\delta MI^{\prime }$-screening on $\delta MI^{\prime }(t)$ and $MI^{\prime }(t)$, Figs. 14(a) and 14(b) present histograms of $\delta MI^{\prime }$ and $MI^{\prime }$ generated from ∼2800 data points across 30 repeatability tests, with and without the $\delta MI^{\prime }$-screening, respectively. Note that the y-axes of Figs. 14(a) and 14(b) are presented on a logarithmic scale to enhance the visibility of outlier values and that $\delta {MI^{\prime }}_{\lim}=10\times 10^{-3}$ of threshold and n=0.5 of power exponent in α-correction have been applied, as is the case in Fig. 13. Moreover, the standard deviation of each histogram, indicated by STD, is presented in the corresponding plot. Looking at each of Figs. 14(a) and 14(b), it was demonstrated that $\delta MI^{\prime }$-screening can effectively remove outlier values in $\delta MI^{\prime }$, thereby reducing the standard deviation of $\delta MI^{\prime }$, and resulting in eliminating inappropriately large $MI^{\prime }$ values.

### 3.4 Repeatability Test Results

**Fig. 15:**
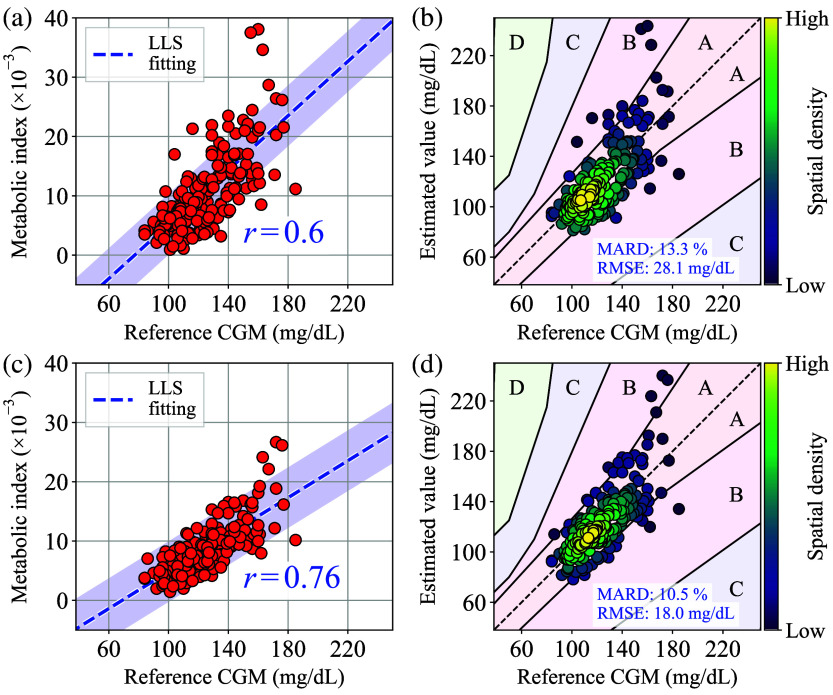
(a) Scatter plot of the obtained $MI^{\prime }$ versus reference CGM values without $\delta MI^{\prime }$-screening. (b) Parkes error grid for type 1 diabetes generated from the results presented in panel (a). (c) Scatter plot of the obtained $MI^{\prime }$ versus reference CGM values with $\delta MI^{\prime }$-screening. (d) Parkes error grid for type 1 diabetes generated from the results presented in panel (c).

Figure 15(a) shows a scatter plot of the acquired $MI^{\prime }(t)$ values versus reference CGM values, generated from the entire repeatability test results, without applying both $\varepsilon_{\sigma }$ and $\delta MI^{\prime }$-screening processes. Here, n=0.5 of power exponent was applied in the α-correction. For illustrative purposes, the linear approximation and the correlation coefficient r calculated by the linear least squares (LLS) fitting are plotted in Fig. 15(a). Furthermore, the highlighted region around the linear approximation indicates a range of $\pm 5\times 10^{-3}$. Figure 15(b) shows a Parkes error grid for type 1 diabetes^27^ that was generated from the results and the conversion coefficients obtained by the LLS fitting presented in Fig. 15(a). Here, each numerical data for $MI^{\prime }(t)$ corresponding to each CGM data point has been calculated by interpolation, resulting in the generation of 183 data point pairs of $MI^{\prime }(t)$ and CGM values. The color scale in the plot indicates the spatial density of nearby points. In this study, CGM values were utilized as the reference values for error grid analysis, in lieu of blood glucose values obtained via venous blood glucose testing. Because of this substitution, note that error grids presented in this paper have combined errors from $MI^{\prime }$ and the CGM. In addition, the mean absolute relative difference (MARD) and the root-mean-square error (RMSE) are also plotted in Fig. 15(b) as BGL-estimation accuracy metrics. Here, in fact, both Figs. 15(a) and 15(b) contain values significantly outside the vertical plot ranges. For reference, Fig. S3 in the Supplementary Material shows zoom-out views of Figs. 15(a) and 15(b).

By applying both $\varepsilon_{\sigma }$- and $\delta MI^{\prime }$-screening to the repeatability test data presented in Fig. 15(a), the scatter plot and the Parkes error grid can be obtained as Figs. 15(c) and 15(d), respectively. Here, the same parameters for $\delta MI^{\prime }$-screening and α-correction applied in Fig. 13, along with the plotting methods employed in Figs. 15(a) and 15(b), were applied to Figs. 15(c) and 15(d). A comparison of Figs. 15(a) and 15(c) reveals that the number of data points within the $\pm 5\times 10^{-2}$ region and the correlation coefficient r are greater when the two-stage screening is applied. In the same way, a comparison of Figs. 15(b) and 15(d) reveals that the number of data points within Zone A and the accuracy metrics are improved by applying the screening process. For reference, zoomed-out views of Figs. 15(c) and 15(d) are shown in Fig. S4 in the Supplementary Material. In addition, Fig. S5 in the Supplementary Material presents a transition movie between without and with the two-stage screening process (Video 1, Mp4, 191 KB [URL: https://doi.org/10.1117/1.JBO.29.10.107001.s1]). Moreover, Figs. S6–S9 in the Supplementary Material illustrate the individual results of the entire oral challenge test.

Finally, Table 1 shows the comparison of key performance metrics with and without the two-stage screening process. For purposes of comparison, the performance metrics observed in the previous research using a smartphone camera-based prototype^9^ are also listed in the table. As demonstrated in Table 1, the smartwatch prototype exhibited comparable or enhanced BGL estimation performance by adopting the proposed two-stage screening process. Moreover, although the BGL range and the number of test subjects are limited and cannot be compared directly, the performance metrics obtained through the proposed screening process are comparable to those obtained through other NIGM methods, including Raman spectroscopy^28^ and radio frequency (RF) spectroscopy.^29^

**Table 1:** Comparison of key performance indicators of BGL estimation with and without screening process.

	Without screening	With screening	Previous research^9^ (smartphone camera)
Zone A percentage	80.3% (147/183)	85.8% (157/183)	78.6% (92/117)
Zone B percentage	18.6% (34/183)	14.2% (26/183)	21.4% (25/117)
Zone C percentage	1.1% (2/183)	0.0% (0/183)	0.0% (0/117)
MARD	13.3%	10.5%	13.3%
RMSE	28.1 mg/dL	18.0 mg/dL	19.7 mg/dL

### 3.5 Parameter Optimization

**Fig. 16:**
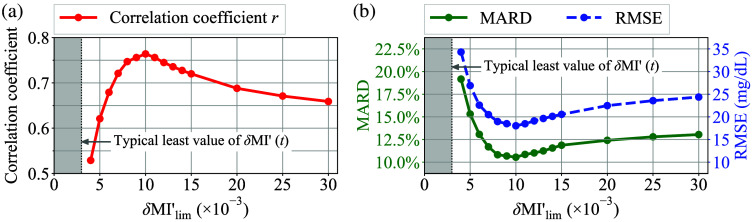
(a) Transition of the correlation coefficient r computed by the LLS fitting according to different $\delta MI^{\prime }$-screening threshold $\delta {MI^{\prime }}_{\lim}$. (b) Transition of MARD and RMSE according to different $\delta {MI^{\prime }}_{\lim}$.

Figure 16(a) shows a transition of the correlation coefficient r computed by the LLS fitting according to different $\delta MI^{\prime }$-screening threshold $\delta {MI^{\prime }}_{\lim}$, with the power exponent n for the α-correction fixed at 0.5. In this plot, the region below a typical least value of $\delta MI^{\prime }(t)$ is filled in gray. Figure 16(a) demonstrates that the correlation coefficient r reaches its maximum value at approximately $x=10\times 10^{-3}$. Figure 16(b) shows a transition of MARD and RMSE according to different $\delta {MI^{\prime }}_{\lim}$. Similar to Fig. 16(a), both MARD and RMSE exhibited their optimal values around $x=10\times 10^{-3}$. In general, a more strict threshold leads to more effective control of data variations. However, in this case, an excessively strict threshold leads to a thorough rejection of the obtained data. This results in insufficient data points in the post-processing, which in turn leads to a deterioration in accuracy. Therefore, it can be inferred that the modest screening threshold exhibited the best performance.

**Fig. 17:**
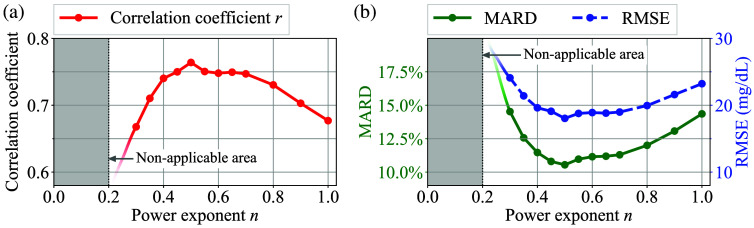
(a) Transition of the correlation coefficient r computed by the LLS fitting according to different power exponent n for the α-correction. (b) Transition of MARD and RMSE according to different power exponents n.

Next, Fig. 17(a) shows a transition of the correlation coefficient r computed by the LLS fitting according to different power exponent n for α-correction, with the threshold for the $\delta MI^{\prime }$-screening fixed at $10\times 10^{-3}$. Here, the area where α-correction is non-applicable due to significant deterioration in accuracy is filled in gray. In this case, the correlation coefficient r attained its optimal value at n=0.5, which implies that the AC amplitude of the optical path length, $L_{\mathrm{AC}}(t)$, is approximately proportional to the square root of the total optical path length, L(t). Similarly, Fig. 17(b) shows a transition of MARD and RMSE according to different power exponents n. The optimal values for MARD and RMSE were observed at n=0.5 in this case as well.

## 4 Discussion

Although 10.5% of MARD has been confirmed by applying the proposed two-stage screening process in Fig. 15(d), it should be noted that this MARD value is equivalent to a training result in the case of the ML method. It is occasionally proposed that only predictive results should be considered in the context of NIGM development.^30^ It is therefore more equitable that the acquired oral challenge results be evaluated by separating them into a training and a test stage.

**Fig. 18:**
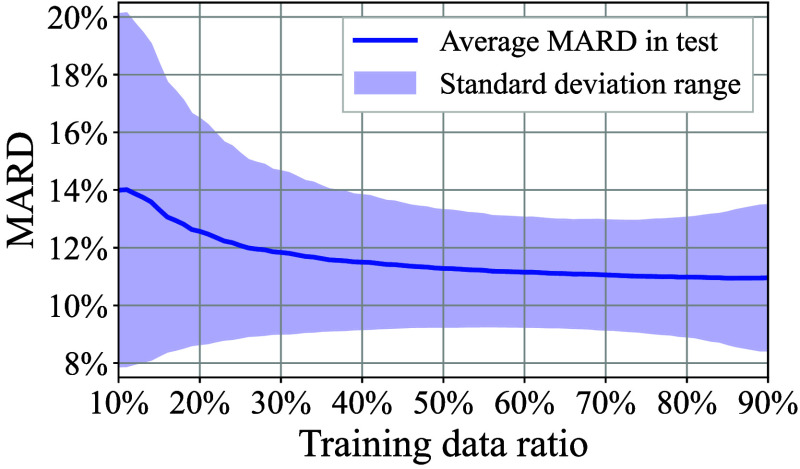
MARD versus the ratio for the training data.

Figure 18 shows the average MARD in the test stage as a function of the specific training data ratio. In the case of a 30% training data ratio, for instance, nine datasets were randomly selected as training data from a total of 30 datasets. The LLS fitting coefficients were calculated from the training data. Subsequently, the MARD was calculated from the remaining 21 datasets for the test stage, using the LLS coefficients that had been derived in the training stage. Given that MARD values in the test stage are affected by the extraction patterns employed for the test data, a sufficient number of repetitions were conducted to ensure the accuracy of the average MARD calculation, utilizing different test data pick-up patterns. In addition, the highlighted region in Fig. 18 indicates the standard deviation range of MARD. Figure 18 indicates that the average MARD is below 12%, which is comparable to the value observed in Fig. 15(d), at 30% of the training data ratio. Then, the average MARD curve exhibits a nearly flat trend above 50% of the training data ratio, accompanied by a minimal standard deviation between 60% and 70% which is a typical training data ratio in a regular ML process. The results demonstrated that the MI method with the proposed two-stage screening process exhibited sufficient performance in the BGL prediction stage as well as the training stage.

**Fig. 19:**
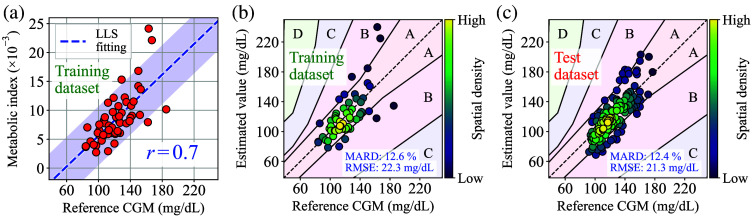
(a) Typical example of the training results at 30% of the training data ratio. (b) Parkes error grid generated from the training result. (c) Test result generated from the learned parameters derived from panel (a).

For reference, Fig. 19(a) shows a typical example of the training result at 30% of the training data ratio. Figure 19(b) shows an error grid analysis result generated from the training result for Fig. 19(a). In addition, Fig. 19(c) illustrates a test result generated from the learned parameters derived from Fig. 19(a).

Here, given that the reference CGM has typically exhibited a MARD of approximately or below 10%, it can be reasonably assumed that 11±2% of the average MARD observed in Fig. 18 at 70% of the training data ratio is nearly equal to the reachable limit under the current experimental protocol. To examine the more precise performance of the MI method, venous blood draw or finger prick SMBG must be adopted as the reference BGL values.

As may be seen in Table 1, the smartwatch prototype exhibited 28.1 mg/dL of RMSE without the proposed two-stage screening process, which represents a 1.5-fold increase in error relative to the smartphone camera-based prototype observed in the previous research. This gap in RMSE can be attributed primarily to the difference in SNR between the prototypes. Specifically, the smartphone camera prototype employs a high-sensitivity CMOS camera with 108 megapixels, whereas the active area of the PD sensor on the smartwatch is optimized for the detection of HR and the measurement of oxygen saturation. Moreover, the LEDs in most commercially available smartwatches draw the optimum current for battery conservation. These factors collectively contribute to the observed difference of over 20 dB in SNR between the two types of prototypes. This ultimately led to a rise in the RMSE of the smartwatch prototype. Although it is demonstrated that this specific disadvantage of smartwatches in the SNR can be compensated through the proposed two-stage screening process, this screening approach is not a fundamental solution. In this study, 53±11% of acquired $\Delta \theta_{\mathrm{FFT}}$ data were rejected through the two-stage screening process in each of the 30 oral challenge tests. In the context of lower ambient temperatures or reduced peripheral blood flow for various reasons, it is anticipated that the data rejection rate will increase, resulting in the creation of multiple void sections in the continuously monitored NIGM data. To address the fundamental issue of smartwatches, it is necessary to consider the enhancement of SNR, particularly in relation to the deoxyhemoglobin NIRS signal. As previously stated, an increase in LED current will result in a reduction in battery life and may potentially lead to an increase in the risk of optical skin burn. Consequently, it would be preferable to pursue an approach that enhances the photodetector in this instance. Indeed, in the case of the smartphone camera-based prototype, which exhibited an SNR that is 10 times or 20 dB superior to that of the smartwatch prototype, the device is largely free from the potential effects of background noise. For the smartphone prototype, the sampling frequency-rooted error represents the most significant contributing factor in reducing noise.

**Fig. 20:**
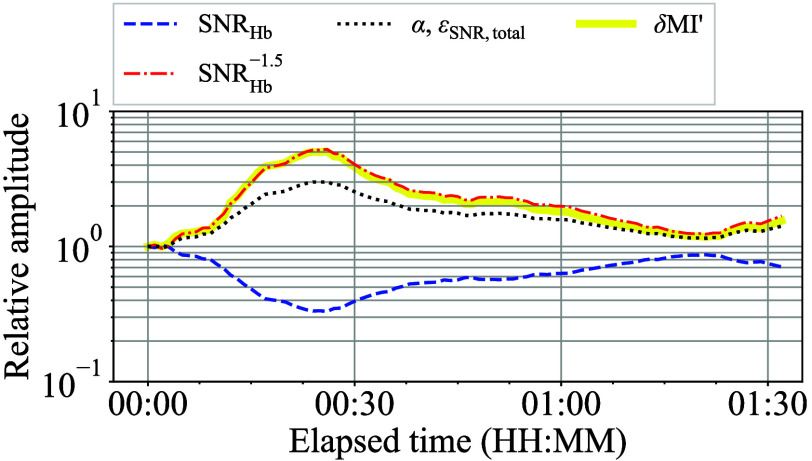
Typical transition of the normalized ${\mathrm{SNR}}_{\mathrm{Hb}}(t)$, α(t), $\varepsilon_{\mathrm{SNR},\text{total}}(t)$, and $\delta MI^{\prime }(t)$ during an oral challenge test.

Figure 20 shows a typical transition of the ${\mathrm{SNR}}_{\mathrm{Hb}}(t)$, α(t), $\varepsilon_{\mathrm{SNR},\mathrm{total}}(t)$, and $\delta MI^{\prime }(t)$ without the proposed screening process, all of which have been normalized at t=0, and this plot is derived from a specific oral challenge test. Note that the y-axis is a logarithmic scale. Figure 20 illustrates that the ${\mathrm{SNR}}_{\mathrm{Hb}}(t)$ reached its lowest value at ∼25  min due to a reduction in the amplitude of the NIRS signal pulsation. Then, according to Eq. (13) with n=0.5 and Eq. (24), α(t) and $\varepsilon_{\mathrm{SNR},\text{total}}(t)$ increased in inverse proportion as ${\mathrm{SNR}}_{\mathrm{Hb}}(t)$, resulting from a lower NIRS signal pulsation amplitude. Finally, $\delta MI^{\prime }(t)$ exhibited an increasing trend at a rate proportional to the inverse of the ${\mathrm{SNR}}_{\mathrm{Hb}}(t)$, to the power of greater than one. For purposes of comparison, the ${\mathrm{SNR}}_{\mathrm{Hb}}^{-1.5}(t)$ curve is plotted in Fig. 20.

As illustrated in Fig. 20, $\delta MI^{\prime }(t)$ may increase by a factor of five times its initial value. This result indicates that $\delta MI^{\prime }(t)$ may reach as high as $25\times 10^{-3}$ even if its typical value at the normal condition is $5\times 10^{-3}$. Therefore, to ensure practical use, the ${\mathrm{SNR}}_{\mathrm{Hb}}$ must have an ample safety margin in place, if applying the NIGM system to smartwatches without the proposed screening process. Given that the typical ${\mathrm{SNR}}_{\mathrm{Hb}}(t)$ value of the smartwatch prototype in a normal condition is ∼50 or 34 dB in decibels, then at least two or even a four times better ${\mathrm{SNR}}_{\mathrm{Hb}}(t)$ equivalent to 40 to 46 dB in decibels is preferable. With 40 dB of typical ${\mathrm{SNR}}_{\mathrm{Hb}}(t)$, the typical $\varepsilon_{\mathrm{SNR},\text{total}}$ will be only 7 mrad, which is approximately one-tenth of the typical $\varepsilon_{s,\text{total}}(t)$ in a 100 Hz sampling state.

In consideration of the form factor of smartwatches, it is not a viable solution to expand the active region of photodiodes by a factor of 4 while maintaining the conventional background noise level.^12^ It is therefore necessary to consider enhancing the photoelectric conversion efficiency and reducing the thermal noise at the amplifier circuit, in addition to increasing the photodiode size as much as possible. In light of the above-mentioned issues, it can be reasonably deduced that augmenting the sampling frequency in the absence of an accompanying SNR enhancement would prove to be an ineffective strategy, particularly given the prevailing circumstances about smartwatches where the SNR represents the dominant source of the BGL estimation error.

Here, on the other hand, it is necessary to discuss the acceptable low SNR threshold. In cases where the α-correction is not a requisite component, or namely, when the optical path length is maintained nearly constant or compensated for through the implementation of suitable opto-mechanical measures, $\delta MI^{\prime }(t)$ can be suppressed to some extent, and the SNR requirement can be eased. Given that α(t)=1, in accordance with the typical conditions for the smartwatch-based prototype, it is possible to maintain $\delta MI^{\prime }(t)$ below 10 mrad with a minimum ${\mathrm{SNR}}_{\mathrm{Hb}}(t)$ of 10, or 20 dB in decibels, which is five times lower than the typical ${\mathrm{SNR}}_{\mathrm{Hb}}(t)$. We can therefore propose with reasonable certainty that implementing opto-mechanical measures for the purpose of compensating the optical path length will provide an effective alternate solution for improving the SNR.

Future studies for more effectively applying the proposed data screening method should also include investigations made by testing multiple subjects with different characteristics such as age, sex, body mass index, and BGL.

## 5 Conclusion

This study presents an analytical derivation of an SQI for the detection of tangible noise on NIRS signals and an investigation of the factors that may affect BGL estimation accuracy. Subsequently, a two-stage data screening process was proposed, utilizing the derived SQI and identified error factors. The effectiveness of this process was validated through 30 oral challenge tests. The implementation of the proposed screening process has led to an enhancement in the accuracy of BGL estimation for the smartwatch-based prototype, which SNR is constrained by the device’s form factor. The proposed screening process would facilitate the integration of wearable and continuous BGL monitoring into size- and SNR-limited devices such as smartwatches and smart rings.

In future studies, to reduce the data rejection ratio through the proposed screening process and enhance data utilization, it is essential to consider making fundamental improvements to the SNR. This can be achieved by combining active area enhancement of photodetectors along with reducing the noise in the amplifier circuit.

## Supplementary Material





## Data Availability

The data underlying the results presented in this paper are not publicly available at this time due to privacy and ethical concerns but may be obtained from the authors after making a reasonable request.
