# Supplementary material for: Accuracy enhancement of metabolic index-based blood glucose estimation with a screening process for low-quality data
Source: J Biomed Opt. 2024 Oct 25;29(10):107001. doi: 10.1117/1.JBO.29.10.107001 (PMC11503645; doi:10.1117/1.JBO.29.10.107001)
Supplement: Supplementary file 1 [file JBO_029_107001_SD001.pdf]

Supplementary Material

S1 Detailed explanation for the derivation of the reachable standard deviation limit

This supplementary section explains the detailed derivation process for the standard deviation limit with a given value for phase delay $\Delta\theta$.

To begin with, let us assume for the sake of simplicity that the BPF-applied oxy- and deoxyhemoglobin NIRS waveforms can be expressed as a continuous function of time t as follows:

$$P(t) = P_0 \sin \omega t, \tag{S1}$$
$$Q(t) = Q_0 \sin (\omega t - \Delta\theta), \tag{S2}$$

where P_0 and Q_0 are the amplitudes of oxy- and deoxyhemoglobin NIRS waveforms, respectively, and ω is the angular frequency of the heartbeat. Here, P_0, Q_0, and the phase delay $\Delta\theta$ are assumed treatable as constants within a limited measurement period. By dividing Eqs. (S1) and (S2) by their amplitudes, normalized oxy- and deoxyhemoglobin waveforms $p(t)$ and $q(t)$ can be obtained as follows:

$$p(t) = \sin \omega t, \tag{S3}$$
$$q(t) = \sin (\omega t - \Delta\theta). \tag{S4}$$

In this context, the variance between the two continuous functions of $p(t)$ and $q(t)$, which is denoted as σ^2, can be expressed with integral operation as follows:

$$\sigma^2 = \frac{1}{T}\int_{t_0}^{T+t_0} [p(t) - q(t)]^2 \mathrm{d}t, \tag{S5}$$
$$= \frac{1}{T}\int_{t_0}^{T+t_0} [\sin \omega t - \sin (\omega t - \Delta\theta)]^2 \mathrm{d}t, \tag{S6}$$

where t_0 is an arbitrary constant, and T is the cardiac cycle which satisfies $T = 2\pi/\omega$. Here, by applying the following sum to product formula of the trigonometric function:

$$\sin A - \sin B = 2 \cos \frac{A+B}{2} \sin \frac{A-B}{2}, \tag{S7}$$

Eq. (S6) can be transformed as follows:

$$\sigma^2 = \frac{1}{T}\int_{t_0}^{T+t_0} [2 \cos (\omega t - \frac{\Delta\theta}{2}) \cdot \sin \frac{\Delta\theta}{2}]^2 \mathrm{d}t \tag{S8}$$
$$= \frac{4}{T} \cdot \sin^2 \frac{\Delta\theta}{2} \int_{t_0}^{T+t_0} \cos^2 (\omega t - \frac{\Delta\theta}{2}) \mathrm{d}t. \tag{S9}$$

By solving Eq. (S9),

$$\sigma^2 = \frac{4}{T} \cdot \sin^2 \frac{\Delta\theta}{2} \cdot \frac{T}{2} \tag{S10}$$

$$= 2\sin^2 \frac{\Delta\theta}{2} \tag{S11}$$

is derived. Finally, by applying the following double-angle formula:

$$\sin^2 \frac{A}{2} = \frac{1 - \cos A}{2} \tag{S12}$$

to Eq. (S11) and then taking the square root, the theoretical standard deviation σ bounded by $\Delta\theta$ is derived as

$$\sigma = \sqrt{1 - \cos \Delta\theta}, \quad (\sigma \geq 0). \tag{S13}$$

S2 Derivation of the standard phase estimation error caused by the background noise

This supplementary section explains the detailed derivation process of the phase estimation error caused by the background noise.

The phase estimation error $\varepsilon_{\mathrm{SNR}}$ can be obtained by solving the following formulas:

$$\varepsilon_{\mathrm{SNR}}^2 = \frac{1}{2\pi} \int_{-\pi}^{\pi} \psi_{\mathrm{SNR},\phi}^2 \mathrm{d}\phi, \tag{S14}$$

$$\psi_{\mathrm{SNR},\phi} = \tan^{-1}\left(\frac{\nu \sin\phi}{1 + \nu\cos\phi}\right), \tag{S15}$$

$$\nu = \frac{1}{\mathrm{SNR}}. \tag{S16}$$

In this context, SNR in Eq. (S16) is defined as the ratio of the amplitude of the main peak intensity to that of the noise floor level, and SNR can be treated as a constant within a limited measurement period. Since Eq. (S14) cannot be solved analytically as it is, several approximations will be introduced to $\psi_{\mathrm{SNR},\phi}$. Firstly, from the perspective of the symmetry of Eq. (S15), Eq. (S14) can be rewritten as

$$\varepsilon_{\mathrm{SNR}}^2 = \frac{1}{\pi} \int_{0}^{\pi} \psi_{\mathrm{SNR},\phi}^2 \mathrm{d}\phi. \tag{S17}$$

Secondly, it can be assumed that the value of ν is sufficiently smaller than 1, which implies that the background noise level is sufficiently smaller than the main peak amplitude. Consequently, Eq. (S15) can be approximated as follows:

$$\psi_{\mathrm{SNR},\phi} \approx \tan^{-1}\Big[\nu\sin\phi \cdot (1 - \nu\cos\phi)\Big], \tag{S18}$$

$$= \tan^{-1}\Big(\nu\sin\phi - \nu^2 \sin\phi\cos\phi\Big), \tag{S19}$$

$$\approx \tan^{-1}\big(\nu\sin\phi\big). \tag{S20}$$

Here, since $\sin\phi$ is at most 1 in $0 \leq \phi \leq \pi$ and ν is sufficiently smaller than 1, Eq. (S20) can be further approximated as follows:

$$\psi_{\mathrm{SNR},\phi} \approx \nu \sin\phi. \tag{S21}$$

For reference, Figure S1(a)-S1(d) shows the comparison of $\psi^2_{\mathrm{SNR},\phi}$ with and without approximation for several values of ν. Looking at Figure S1(b), the approximated function is close enough to the original $\psi_{\mathrm{SNR},\phi}$ when $\nu = 0.1$. Further, looking at Figure S1(c) and S1(d), the approximated function is almost identical to the original $\psi_{\mathrm{SNR},\phi}$ when ν is smaller than 0.05.

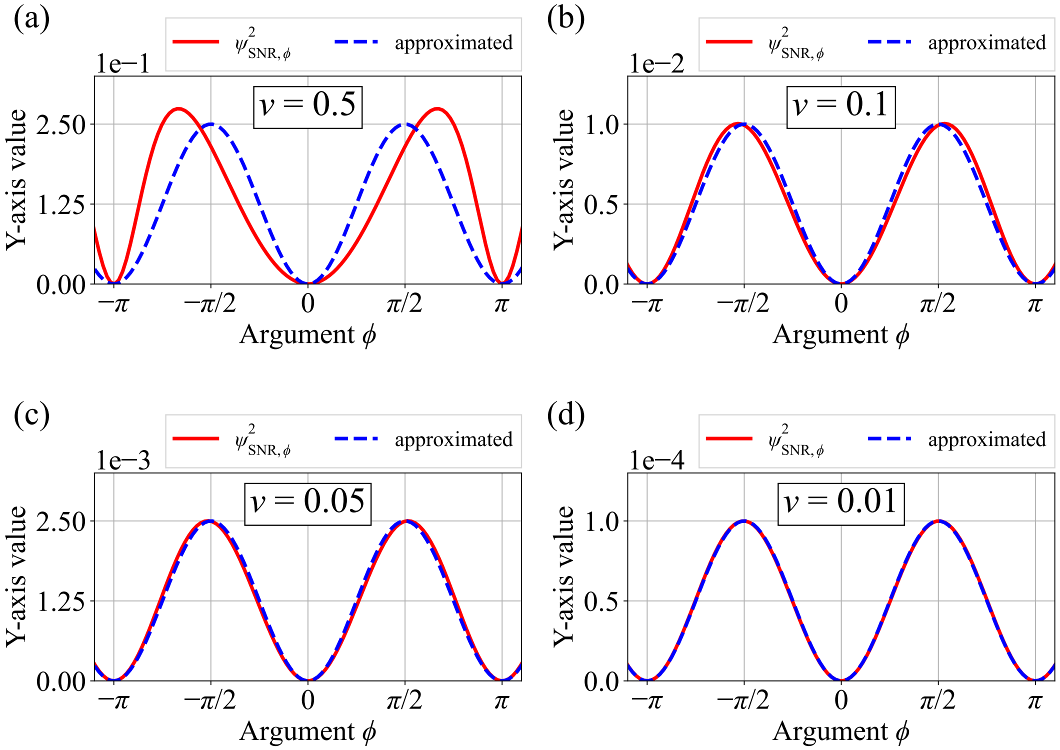

Fig S1 (a)-(d) Comparisons between the functions $\psi^2_{\mathrm{SNR},\phi}$ with and without approximation and with different ν values.

Then, by substituting Eq. (S21), Eq. (S17) is expressed and calculated as follows:

$$\varepsilon^2_{\mathrm{SNR}} \approx \frac{1}{\pi}\int_0^{\pi} \nu^2 \sin^2\phi \mathrm{d}\phi, \tag{S22}$$

$$= \frac{1}{\pi}\cdot\nu^2\cdot\frac{\pi}{2} = \frac{\nu^2}{2}. \tag{S23}$$

Finally, by applying Eq. (S16) and taking the square root to Eq. (S23), $\varepsilon_{\mathrm{SNR}}$ is obtained as

$$\varepsilon_{\mathrm{SNR}} \approx \frac{1}{\sqrt{2}}\cdot\frac{1}{\mathrm{SNR}}. \tag{S24}$$

S3 Table of key nutrition facts in oral challenges

Table S1 shows a list of the main nutritional values in the oral challenges.

Table S1 Key nutritional facts in oral challenges per serving.

Item	Coca-Cola® Original	in Jelly® Energy
Serving size	350 mL	180 g
Calories (kCal)	140	180
Fat (g)	0	0
Carbohydrate (g)	39	45
Protein (g)	0	0

S4 Supplementary plots for experimental results

S4.1 Transition of binarized results

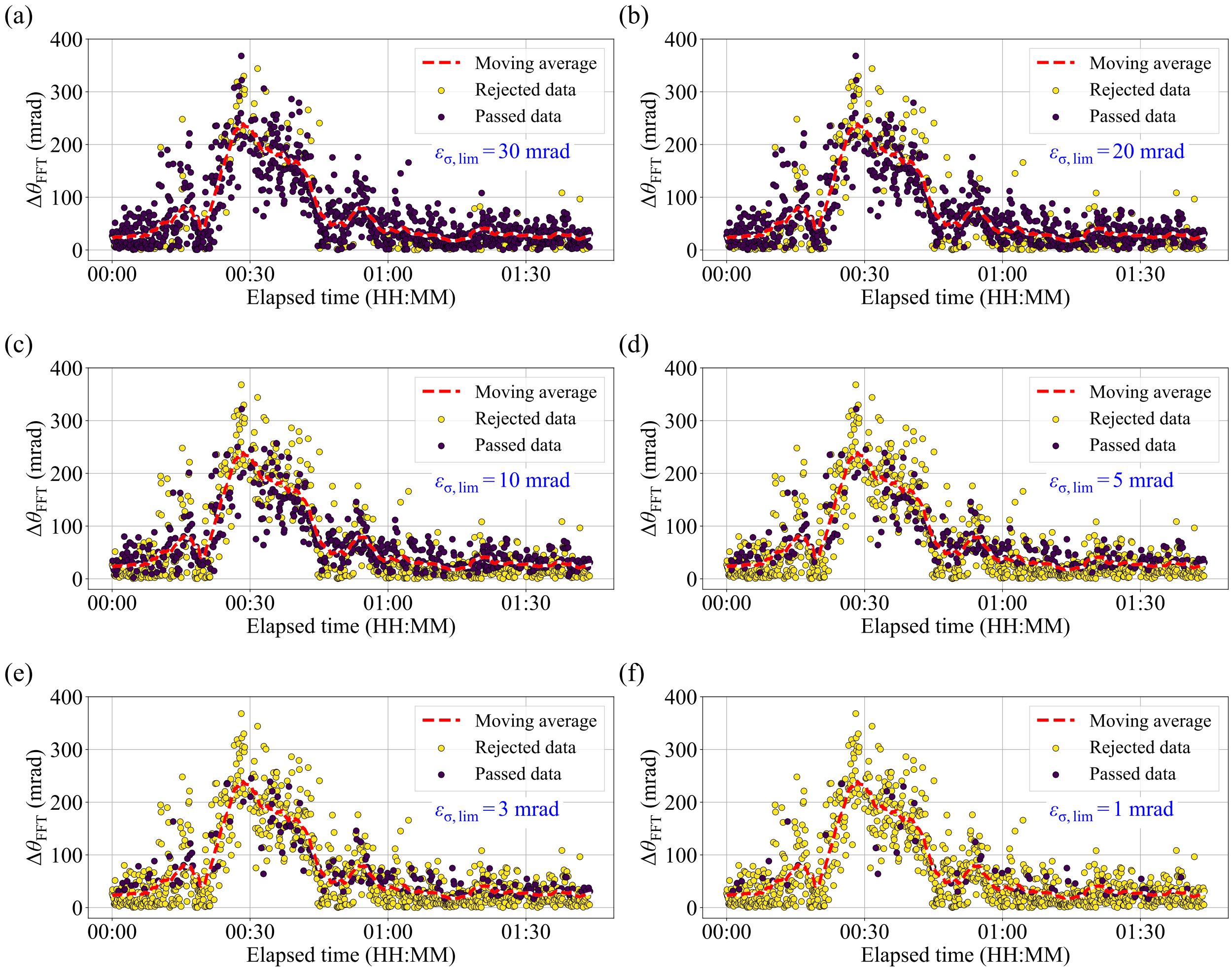

Fig S2 (a)-(d) Transition of binarized results of ε_{σ}-screening for various $\varepsilon_{\sigma,\mathrm{lim}}$ values.

S4.2 Zoomed-out views of repeatability test results

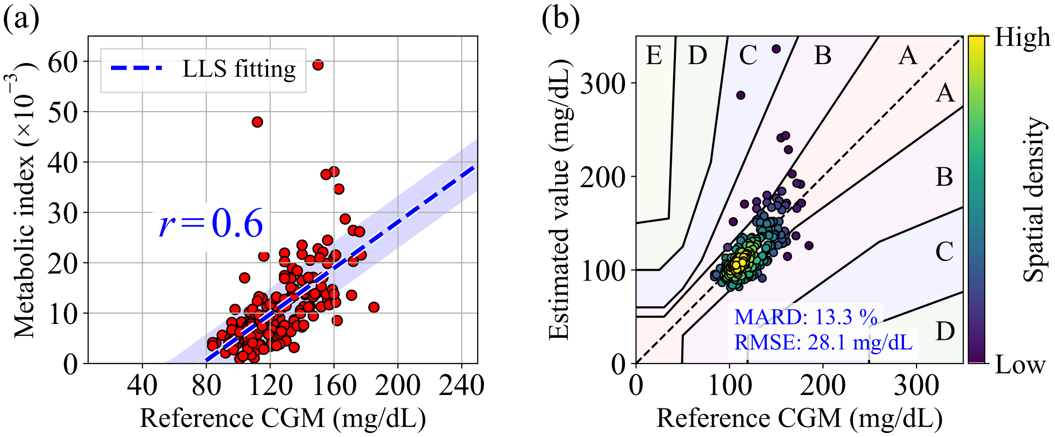

Fig S3 Zoomed-out view of the repeatability test results without the two-stage screening process.

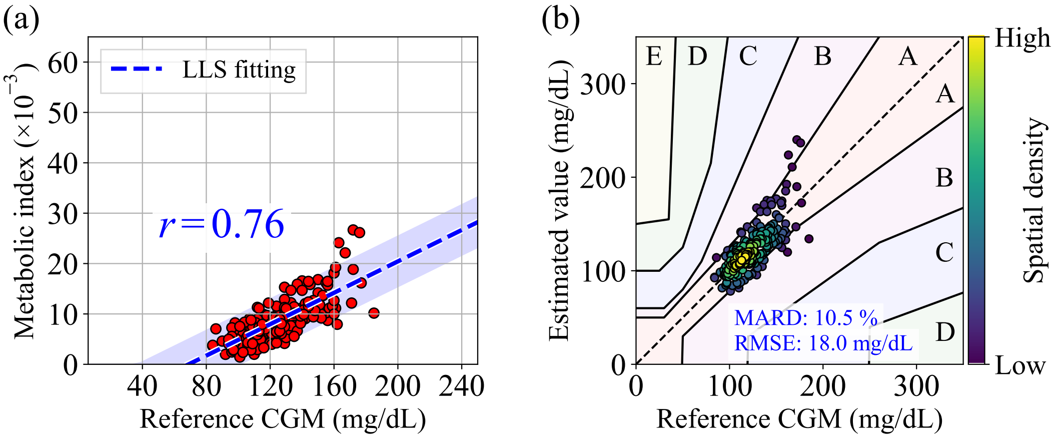

Fig S4 Zoomed-out view of the repeatability test results with the two-stage screening process.

S4.3 Video demonstrating the transition from Figure S3 to Figure S4.

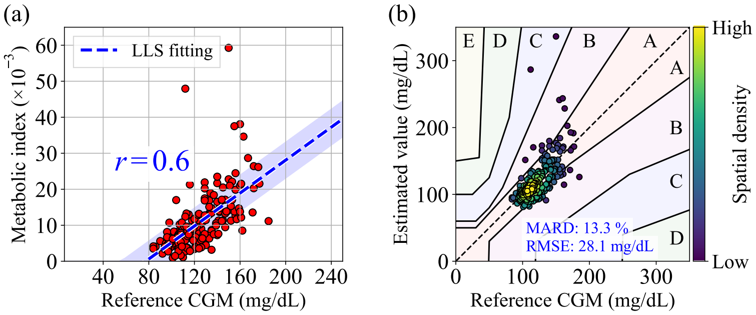

Fig S5 Transition between without and with the two-stage screening process. (Video 1, MP4, 0.2 MB)

S4.4 Individual results of the repeatability test with the two-stage screening process

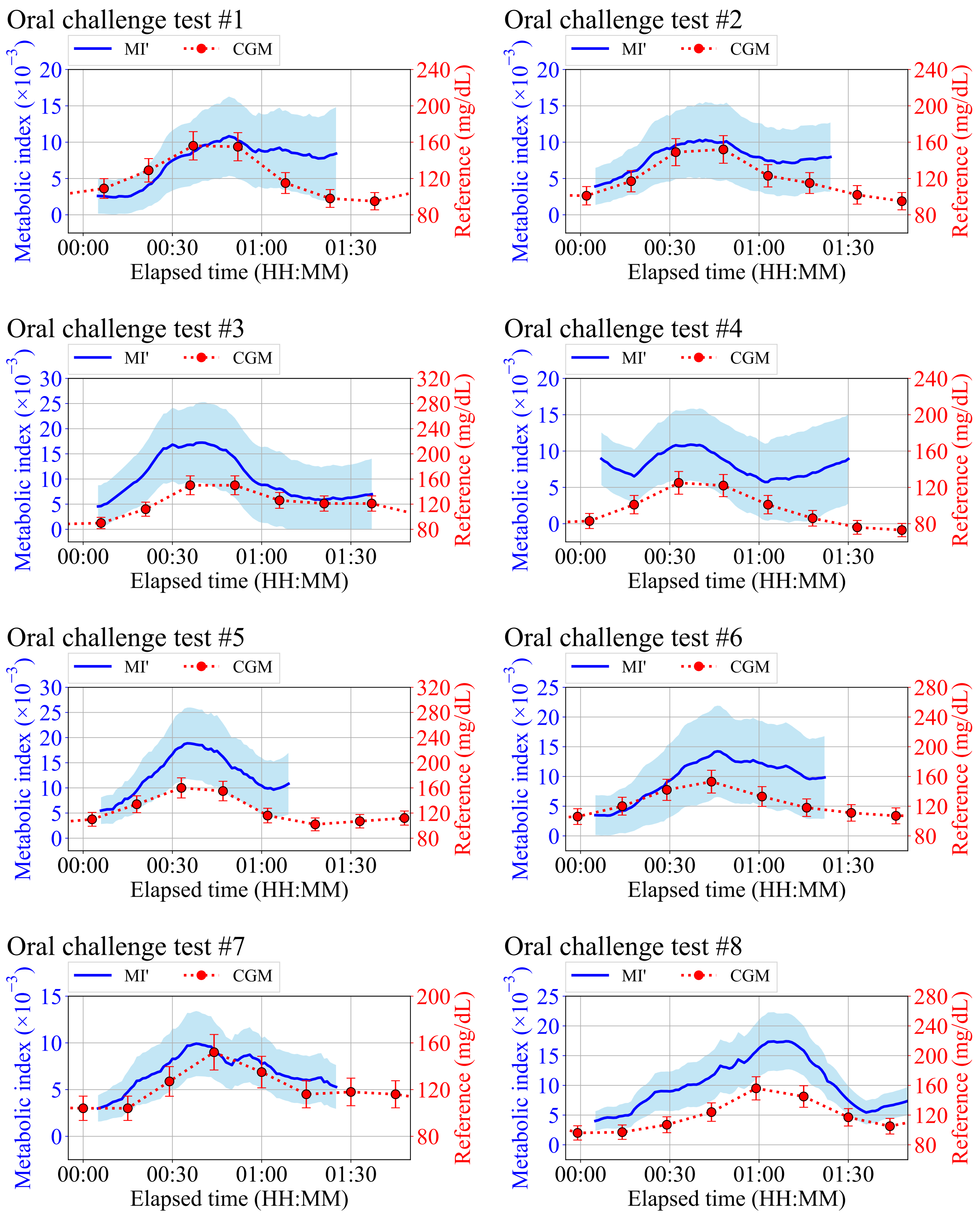

Fig S6 Individual results of the repeatability test with the two-stage screening process. (1 of 4)

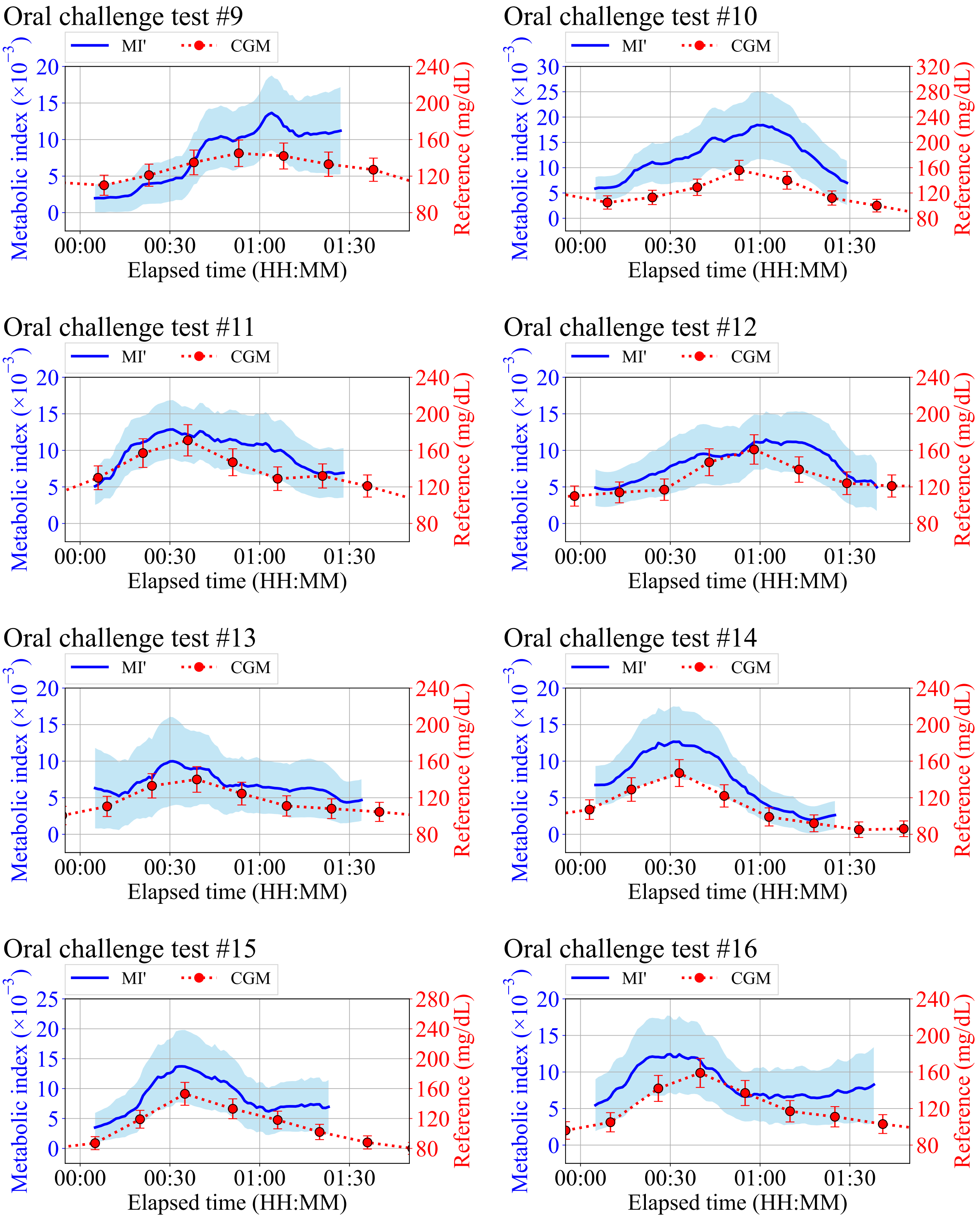

Fig S7 Individual results of the repeatability test with the two-stage screening process. (2 of 4)

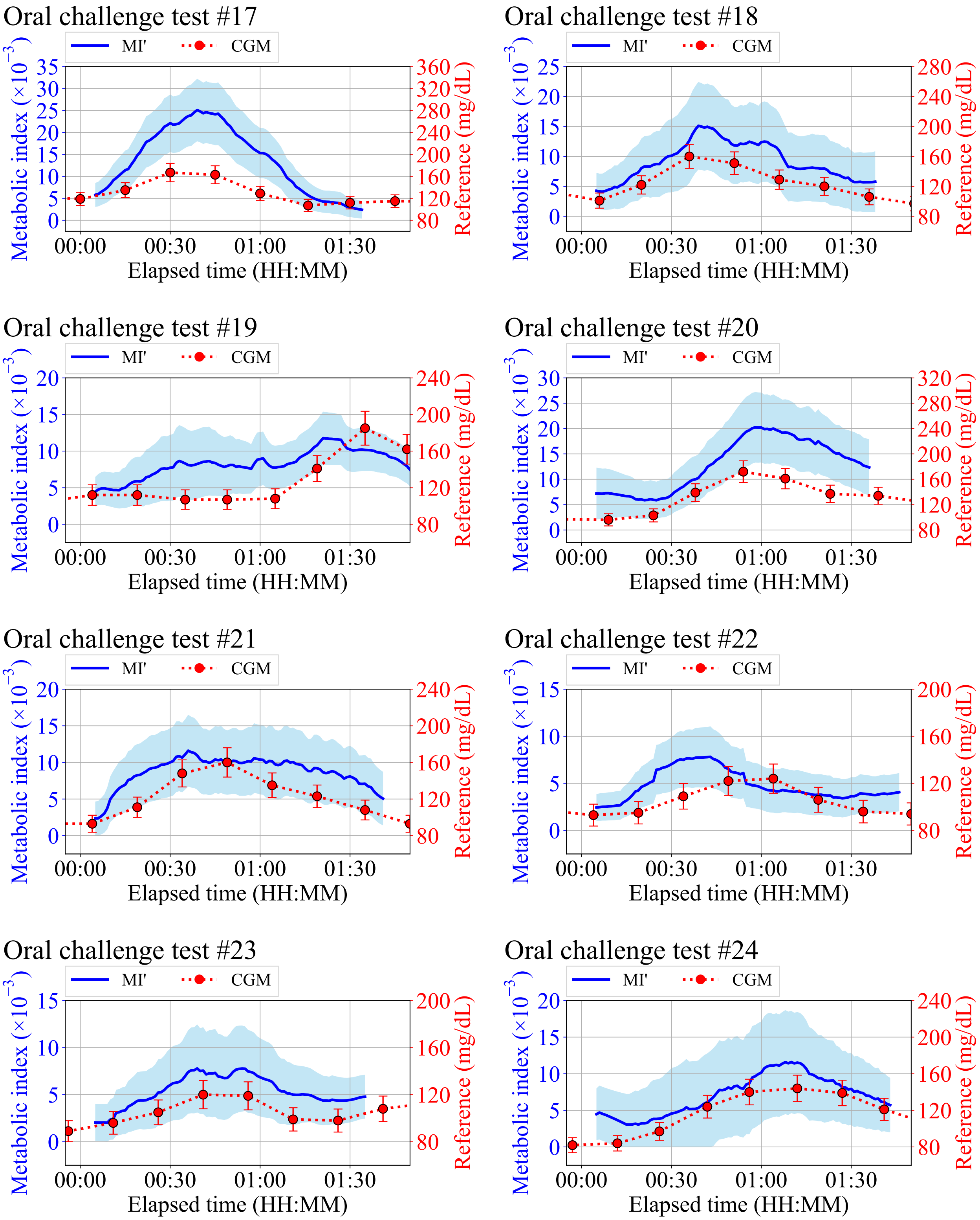

Fig S8 Individual results of the repeatability test with the two-stage screening process. (3 of 4)

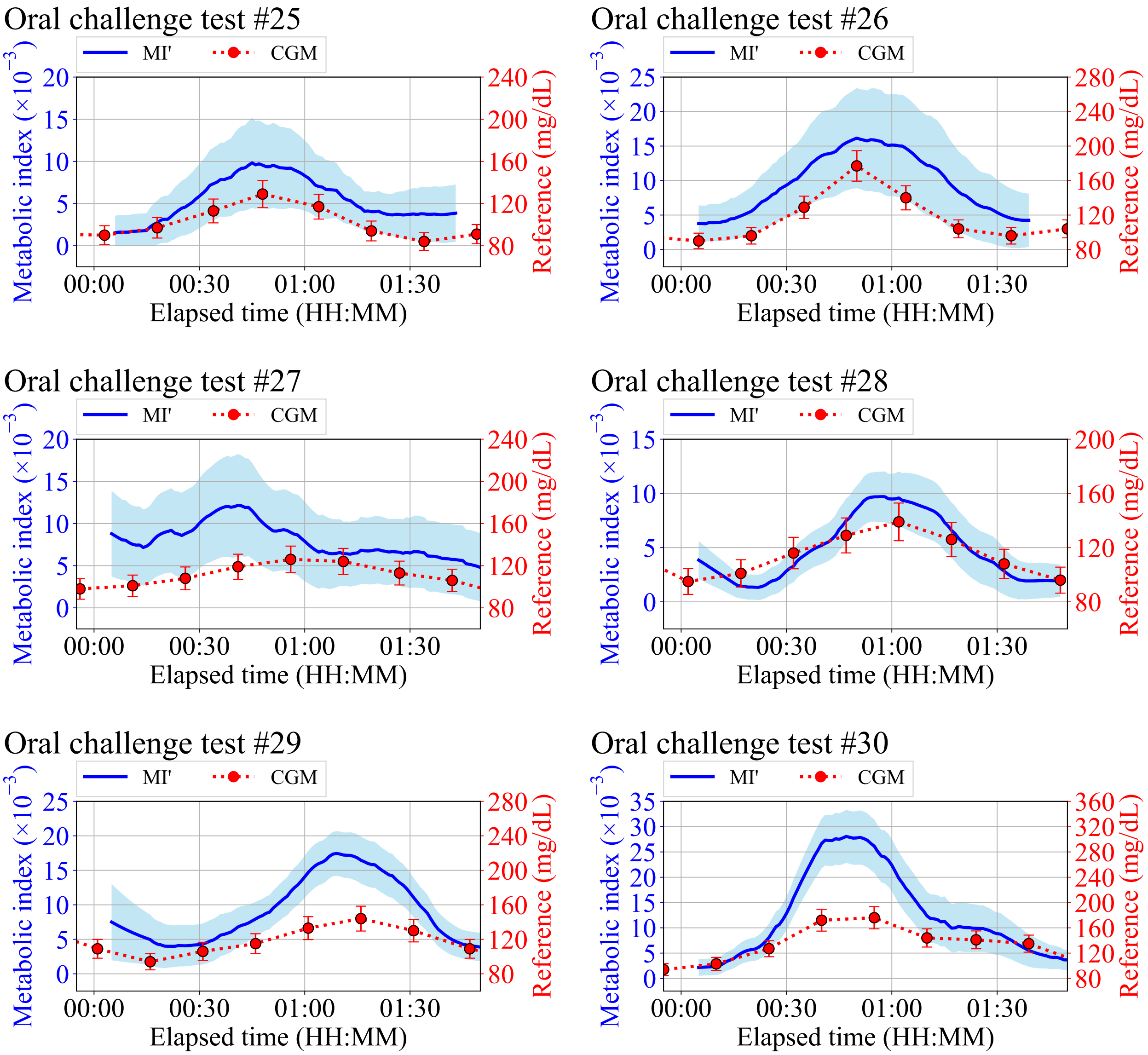

Fig S9 Individual results of the repeatability test with the two-stage screening process. (4 of 4)

S5 Appendix

Table S2 shows a list of abbreviations used in the article.

Table S2 List of abbreviations

Abbreviation	Meaning
BGL	Blood Glucose Level
BPF	Band-Pass Filter
CGM	Continuous Glucose Monitoring
FDA	The United States Food and Drug Administration
FFT	Fast Fourier Transform
HR	Heart Rate
HRV	Heart Rate Variability
LLS	Linear Least Squares
MARD	Mean Absolute Relative Difference
MBLL	Modified Beer-Lambert Law
MI	Metabolic Index
ML	Machine Learning
MNA	Motion and Noise Artifact
NIGM	Non-Invasive Glucose Monitoring
NIR	Near Infrared
NIRS	Near Infrared Spectroscopy
OTC	Over-the-Counter
PPG	Photoplethysmography
RF	Radio Frequency
RMSE	Root-Mean-Square Error
SMBG	Self-Monitoring Blood Glucose
SNR	Signal-to-Noise Ratio
SQI	Signal Quality Index
